# Which emphasis technique to use? Perception of emphasis techniques with varying distractors, backgrounds, and visualization types

**DOI:** 10.1177/14738716211045354

**Published:** 2021-09-22

**Authors:** Aristides Mairena, Carl Gutwin, Andy Cockburn

**Affiliations:** 1 University of Saskatchewan, Saskatoon, SK, Canada; 2 University of Canterbury, Christchurch, New Zealand

**Keywords:** Data visualization, perception, empirical evaluation, experimental studies, human-computer interaction

## Abstract

Emphasis effects are visual changes that make data elements distinct from their surroundings. Designers may use computational saliency models to predict how a viewer’s attention will be guided by a specific effect; however, although saliency models provide a foundational understanding of emphasis perception, they only cover specific visual effects in abstract conditions. To address these limitations, we carried out crowdsourced studies that evaluate emphasis perception in a wider range of conditions than previously studied. We varied effect magnitude, distractor number and type, background, and visualization type, and measured the perceived emphasis of 12 visual effects. Our results show that there are perceptual commonalities of emphasis across a wide range of environments, but also that there are limitations on perceptibility for some effects, dependent on a visualization’s background or type. We developed a model of emphasis predictability based on simple scatterplots that can be extended to other viewing conditions. Our studies provide designers with new understanding of how viewers experience emphasis in realistic visualization settings.

## Introduction

Emphasis effects are visual changes that make important data elements distinct from their surrounding context. Emphasizing elements in a visualization to direct a viewer’s attention or to indicate importance is an essential practice in visualization design. Designers may use emphasis to encourage exploration (e.g. by highlighting regions of interest to signify importance or to alert viewers to missing links); and in narrative visualization, when known aspects of a data set are presented to the viewers,^1,2^ designers may alter an element’s size to improve its legibility relative to other areas of a visualization. A common feature of these techniques is that they bring a viewer’s visual focus to a particular prominent element.

For emphasis to be effective, highlighted elements should be visually distinct from the surrounding context; thus, designers of information displays need to know how well an emphasis effect will work. A wide variety of visual effects have been found to “pop out”– that is, they are immediately distinguishable due to their unique visual properties, allowing them to create clear visual emphasis.^3,4^

Emphasized elements can be quickly identified by a user because their unique visual features guide visual attention. The human visual system can pre-process visual features in an entire scene without conscious attention,^5,6^ and elements that pop out can be rapidly identified,^3,5–8^ independent of the number of elements in a display. Designers may use computational saliency models to predict how a viewer’s attention will be guided by a specific effect^9^; however, although saliency models provide a foundation for understanding perception of emphasis, they only cover specific visual effects in abstract conditions. Metrics for predicting perceived differences in visual channels come from controlled models of human vision, but real-world visualizations are complex and can be viewed on a wide range of devices and in a wide range of environments.

Recent theoretical frameworks of perceived emphasis link visually diverse emphasis effects through the idea of visual prominence, and classify emphasis effects in terms of several basic factors. For example, an *intrinsic* emphasis effect provides prominence that arises from differences in the visual representation of the data points; an *extrinsic* emphasis effect increases visual prominence using an additional visual variable such as color highlighting applied to an existing visual representation.^10^ Frameworks also differentiate between *time-variant* effects (i.e. those that change over time, such as flicker or motion) and *static* effects that do not vary as time progresses.^10^

It is critical for designers to understand how the emphasis effects that they use in a visualization will be perceived by users: they need to know whether the emphasis effect will reliably draw users’ attention (and how quickly), and they need to know whether users will understand that the effect is in fact indicating emphasis (i.e. some effects may draw the user’s visual attention but without the user realizing that the emphasized element is important). Although several studies have been carried out to explore different aspects of how emphasis is perceived, previous work is limited to a small number of visual variables and a small number of viewing contexts. Therefore, one of our primary goals in this research is to substantially expand on the visual combinations that have been tested: in the studies described below, we quantify the influence of a variety of arrangements and visualizations to derive the perceptibility limits of a wide range emphasis effects. This extension of previous studies is important because subtle changes to a visualization can have large effects on emphasis (e.g. Duncan and Humphreys^11^ show that even slight variation in distractors decreases performance) – this and other work clearly shows that designers need a broader understanding of how emphasis will work in realistic information settings.

To address the limitations of current models and available evaluations, we carried out a series of crowdsourced studies that evaluate emphasis in a wider range of conditions than have previously been studied. We studied 12 different visual effects (including static, time-varying, intrinsic, and extrinsic effects), and varied effect magnitude, distractor number and type, background type, and visualization type; for each combination, we measured users’ ability to identify that an element was emphasized, as well as their subjective ratings of the degree of emphasis. Furthermore, we tested several combinations that have not been seen in previous work: for example, we test differences between static and time-varying effects, between different types of time variance (changing the duration of the effect vs changing the magnitude), and between filled and unfilled targets.

Our results show that there are commonalities in perception of emphasis effects across a wide range of environments, including varied visualization types and backgrounds, but also that there are important limitations on perceptibility for some effects:

- *Background manipulations and magnitude affect perceptibility*: There are significant differences in the way visual variables are affected by magnitude levels, clutter, backgrounds, and visualization types. Overall, the magnitude of an effect provides a graduated way to increase or decrease perceived prominence for most variables, but some effects such as motion reached a performance ceiling even at low magnitude levels, but only for simple scatterplots.
- *Clutter affects size perception*: Clutter (i.e. the number of distractors) increased search time for size-based emphasis effects; other effect types, however, were largely unaffected.
- *Increased distractor types affects only certain emphasis effects*: Increasing the types of distractors only reduced the effectiveness of visual variables that were similar to the distractors (e.g. when there were multiple shapes as distractors, shape was less effective as emphasis); however, other visual variables were robust to this change, showing that emphasis effects can still be used in complex visualizations, if they are chosen carefully.
- *Differences between time-variant and time-invariant effects*: We found that time-invariant effects often outperformed time-variant effects, and an analysis of the type of time variance showed that variables that hold a constant time were more successful at indicating emphasis. We additionally found no difference between filled and unfilled shapes.
- *Commonalities among* visualization *types*: As a starting point, we tested emphasis in a range of visualization types and backgrounds, and we found similarities among several visualizations, suggesting that perceptibility results from simple scatterplots can be extended to other visualization types.
- *Interaction between real-world* visualizations *and emphasis*: We observed several specific interactions visualization types and different emphasis effects: for example, some effects were much less visible in Hexbin visualizations (likely because of the space-filling nature of this visualization style).

We also developed a predictive model of emphasis based on magnitude, and our results show that the model can be extended to other visualization types and viewing conditions. Overall, our work substantially expands on previous analyses and models by including a wider range of emphasis effects, and uses a crowdsourced approach that allows our results and model to incorporate a variety of typical viewing conditions. Our studies provide empirical evidence that increases our understanding of how visual emphasis effects are experienced by viewers, in a much wider range of visualization conditions than have previously been studied. Our findings provide valuable information for designers who want to control the user’s visual experience with a variety of emphasis effects in different scenarios.

## Related work

### Saliency and graphical perception

Treisman and Gelade^5^ was one of the first attention researchers to study the nature of pre-attentive processing, focusing on particular visual properties of image features that lead to fast and scale-invariant processing. This work led to the development of the Feature Integration Theory, which suggests that the human visual system maintains a set of feature maps for different visual features such as color or shape. These feature maps are encoded in parallel, leading to almost instantaneous detection, independent of the number of distractors.

Several new theories build on Triesman’s early work (see Wolfe^12^ and Healey^3^ for a review), but a common element among these theories is that attention operates by selecting features of the incoming sensory data for further processing.^13^ For example, the Guided Search Theory suggests a two-stage process for visual attention where attention can be biased toward targets of interest (e.g. a user looking for a red circle) in the top-down phase by encoding particular visual characteristics.^14^ In this two-stage process, the user looking for the red circle assigns a higher weight to the red color. Recent theories also propose that perception prioritizes items that have been previously selected, leading to a three-stage model for visual attention comprising current goals, selection history, and physical salience (bottom-up attention).^15^

An important step for emphasis in visualization is the identification of basic visual variables that can be manipulated to encode information. Much prior research from graphical perception has investigated the degree to which visual variables such as position, length, area, shape, and color aid understanding for a variety of visualization tasks.^16^ The basic properties of visual variables as outlined by Bertin et al.^17^ suggest that varying an element’s visual representation is an effective tool for encoding information and achieving noticeability. Bertin identified seven basic variables to construct visualizations: (1) location, (2) size, (3) color hue, (4) color value, (5) grain, (6) orientation, and (7) shape. Particularly, Bertin suggests that selective visual variables – variables which allow viewers to immediately isolate a group of symbols based on a change in the variable – such as position, size, color hue, or texture enable viewers to quickly detect differences. Following Bertin, researchers have suggested further visual variables such as (9) clarity, (10) resolution, (11) transparency, (12) color saturation, and (13) arrangement.^18,19^

Many visual features also “pop out”– a term used to describe a target item when it is easily identified due to its unique visual properties in visual searches – beyond the initial visual variables identified by Bertin. In a review of the area, Healey and Enns^3^ identified sixteen visual features that have all been successful at creating pop-out effects. Recent studies have also considered questions such as whether popout occurs when the target is composed of a combination of visual features, when targets are in the visual periphery,^4^ whether identification is affected when there are multiple kinds of distractors,^20^ or if popout is affected by common tasks such as switching screens.^21^ Variables that create popout effects are particularly relevant for emphasis because they are detectable in the “pre-attentive” phase of visual processing.

Establishing the effectiveness of visual variables and features in graphical perception tasks is essential for visualization design. For example, Simkin and Hastie^22^ tested value discrimination and estimation for bar, divided bar, and pie charts. Spence and Lewandowsky^23^ use a discrimination task to investigate perception of percentages in bar charts, pie charts, and tables. Various projects investigate shape and motion discrimination in scatterplots.^24–26^ Each of these studies measures how a visual variable (e.g. position, size, hue) affects the accuracy and/or response time of estimating values of the underlying data.^24–27^ Many of the early studies of visual perceptibility were carried out by Bartram and Ware,^8,28^ with results suggesting that simple motion effects are effective at driving attention to notifications in information-dense displays.

In our work, we extend recent efforts in graphical perception for emphasis to explore perceptual difference between visual effects and extend analysis beyond traditional scatterplots to quantify potential differences across visualization types.

### Perception in visualization

Empirical studies of visual variables in visualization have generally evaluated their utility for design and their individual features for guiding visual perception. For example, Smart and Szafir^29^ measure how size and shape affect color perception, Demiralp et al.^30^ developed the idea of “perceptual kernels” to measure the perceived similarities between pairs of visual variables (color, size, and shape), and Haroz and Whitney^31^ explored the perceptual limits of attention for visual effects, investigating how visual feature type (color vs motion), layout, and variety of visual elements affect user performance, with findings suggesting that features that are known to be pre-attentive are adversely affected by arrangements and tasks that also require attention. Similar to both Haroz and Whitney and Demiralp et al., our experiments also address the question of whether a viewer can determine if a target is present on the screen; we expand on their findings by including a much larger number of effects, and explore a set of visualizations taken from the real world to increase our understanding of how layout and visual elements affect performance.

Other studies of visual variables for visualization design have focused on the utility of certain features for conducting analysis: for example, Cleveland and McGill^32^ found that color is less precise at communicating data than other visual variables such as size and position; MacEachren et al.^33^ evaluated the effectiveness of color among a variety of visual variables for communicating uncertainty in visualization; and Boukhelifa et al.^34^ found that differences in certain visual variables such as blur and value are difficult to estimate. Work by Correll et al.^35^ also evaluates perception, but focusing on color ramp design, and shows that manipulating color ramp structures can improve a viewer’s judgment in uncertainty estimation.

Perception studies have continuously explored scatterplots,^36–40^ as they are one of the most effective visualizations for visual judgments because data points are positioned along a common scale.^41^ Work by Li et al.^39,40^ models size perception for analytical tasks in scatterplots, Pandey et al.^38^ models scatter plot similarity according to their visual features (visual features of the underlying data such as correlations, density, groupings, orientations), while Yang et al.^37^ evaluates how readers attend to visual features while performing discrimination tasks using scatterplot visualizations.

Perception in visualization is often evaluated through visual search tasks that measure reaction times and accuracy^42^; these measures serve as proxies for attention based on the premise that search for salient items should be faster and more accurate than search for items that do not draw attention. Early computational saliency models that aimed to predict where a viewer’s attention would be directed were accurate at predicting attention with natural images,^43^ but performed poorly on visualizations.^44^ Visualizations are often designed with abstract dots and lines and contain textual information such as labels and legends which guide the viewer’s attention, making it difficult for saliency models based on natural images to predict attention in visualizations.

To address this limitation, Matzen et al.^45^ introduced a saliency model for visualization which modeled how bottom-up factors influence a user’s visual attention during exploration which was then extended to incorporate top-down features by Polatzek et al.^46^ However, we currently lack models specific to emphasis in visualization. Kong et al.^47^ provide an initial evaluation of the memorabilty and comprehensibility of visual stimuli “in the wild,” but this study is limited to three variables. An initial model of emphasis perception suggested by Mairena et al.^48^ is effective at predicting perception based on simple scatterplots when compared to more complex visualizations, but is similarly limited to three visual variables (color, size, and blur/focus) in a controlled laboratory environment. Our work expands on these evaluations and builds models to take into account a greater variety of settings.

### Emphasis in visualization

The goal of emphasis is to manipulate the visual features of an important data element to make it visually prominent, such that a viewer’s bottom-up attention is attracted to that element.^10^ There are several theories that designers must take into account when designing emphasis. For example, similarity theory shows that visual search efficiency decreases with increased target/non-target similarity and with decreased similarity between the non-targets.^11^ However, if users are given a specific task, or have a feature they are interested in (e.g. a user searching for a red circle), the relational account of attention theory suggests that attention will be guided to the mark that differs in the given direction from the other marks. Additionally, the similarity of all non-targets, and the channel offset (i.e. the lowest value of the non-targets) have also been found to influence perception.^9^

To make a data point prominent and generate emphasis, visual techniques make the focus mark (i.e. the target) sufficiently dissimilar from the other elements (i.e. the non-target marks) in at least one visual channel.^10^ For example, techniques such as blur/focus, magnification, and highlighting create emphasis by making one data point more visually prominent than others (e.g. sharper, bigger, or a different color). Early work on emphasis focused on distortion and magnification techniques,^49–51^ and other techniques such as blur,^52^ motion,^53^ and flicker^54^ have also been identified. To categorize the wide range of visual features for generating emphasis, Hall et al.^10^ suggested a categorization of emphasis effects into two main groups based on how visuals change over time: time-invariant and time-variant effects.

Effects such as highlighting (coloring a data point in a visualization),^55,56^ and blurring (where a data point is shown in focus while the other elements are blurred) are considered time-invariant emphasis effects. In contrast, effects that are achieved through animations and transitions that alter the appearance of a point such as motion, flicker, or pulse are considered as time-variant emphasis effects.

Despite this rich body of research, findings from classic visual search and psychophysics experiments in controlled environments do not directly apply to visualization in practice. Perception of emphasis is affected by many factors such as background luminance,^57,58^ density,^58,59^ chart size,^60^ scale,^61^ or aspect ratio.^60,62^ Additional studies suggest that environmental context may also affect how viewers decode visualized data. These results reinforce the need for empirical evaluations of visual effects in visualizations to validate theory and evaluate real-world visualization applications. Table 1 summarizes the studies that have been carried out on perception of emphasis in visualizations, and clearly shows that there is still relatively limited coverage beyond a few visual variables, environmental manipulations, and visualization types. To address this limitation, we carried out the web-based crowdsourced experiments described below – our studies assess perception of visual prominence and the effectiveness of a variety of emphasis effects at a wide range of intensity levels, viewing conditions, and visualization types.

**Table 1.** Summary of papers according to visual variables and grouped by emphasis types (time variant/time invariant).

	Color	Size/area	Blur/focus	Opacity	QTONs	Shape	Motion	Flicker	Pulse	Other	Vis types	Method	Environment
Mairena et al.^48^	✓	✓	✓								Scatter	L	Gaze-tracking
Li et al.^39^		✓									Scatter	L	
Li et al.^40^		✓				✓					Scatter	L	
Yang et al.^37^		✓				✓				✓	Scatter	C	
Smart and Szafir^29^	✓	✓				✓					Scatter	C	
Demiralp et al.^30^	✓	✓				✓					N/A	C	
Kong et al.^47^			✓	✓		✓				✓	Various	L + C	Gaze-tracking
Veras and Collins^24^	✓	✓					✓				Scatter	C	
Bartram et al.^28^	✓					✓	✓				N/A	L	
Cleveland and McGill^32^	✓	✓								✓	N/A	L	
Haroz and Whitney^31^	✓						✓				Various	L	
Gutwin et al.^4^	✓	✓		✓		✓	✓	✓		✓	N/A	L	Multi-displays
Current Paper	✓	✓	✓	✓	✓	✓	✓	✓	✓		Various	C	

L: lab experiment; C: crowdsourced experiment. N/A visualization type means that no specific visualization was used (e.g. stimuli on an abstract or black background).

## General study methods

Our goal is to understand perception of emphasis in ways that can inform real-world visualization design. This goal underlies the increased number of emphasis effects and visualization settings tested in our studies – but our crowdsourced approach also plays a role in making our results more generalizable, because designers cannot know in advance the user’s environmental factors such as display size, graphics calibration, monitor resolution, or ambient lighting. These factors mean that designs may look different for different users. A recent approach to accounting for this variance that has been used in prior visualization studies is the idea of using a large crowd-sourced sample^24,29,41,63,64^: although crowdsourcing implies higher individual differences due to monitor and browser settings, this variance also covers a wider variety of realistic viewing scenarios, allowing for more general predictions about how viewers will perceive visualizations in the real world – often with higher accuracy than models and results from traditional laboratory studies.

In the following studies, we experimentally evaluate how specific emphasis effects are experienced by a viewer in a wide range of scenarios. Our first study considers the visual prominence of 12 different emphasis effects in simple scatterplots, and our second, third, and fourth studies consider these effects in different viewing conditions. Study 1 determines the baseline visual prominence of 12 emphasis effects that use different visual variables (blur/focus, color, size, pulse, flicker, motion, opacity, and shape). Each effect was tested at six magnitude levels in a typical scatterplot visualization where we emphasized one data element and measured participants’ ability to perceive the emphasis using a binary-choice design that asked participants to state whether they saw an emphasized stimuli; we also measured search time and subjective ratings of visual prominence. Studies 2 and 3 used a similar paradigm but varied the visualization to look for environmental effects on perception of emphasis: Study 2 varied the visualization’s clutter (i.e. number of distractors), and Study 3 varied the types of distractors (e.g. including multiple shapes and colors). Finally, Study 4 varied the type of visualization (moving beyond simple scatterplots to test perception of emphasis with different visualization styles and backgrounds).

### Visualization environment and task

**Figure 1:**
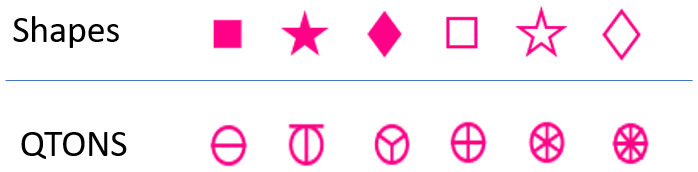
Participant view of the object field in Study 1; target showing size emphasis effect is at lower right. .

All of our experiments use a similar presentation approach and task: a visualization is presented to the participant, in which one data item may be emphasized using one of our visual effects. Similar to previous research,^9,29,42,64^ our studies use a binary forced-choice design, meaning that we asked participants to state whether any element in the visualization appears to be emphasized or not. In some of the visualizations there was no emphasized element, to ensure that participants were maintaining a reasonable level of accuracy (i.e. not just answering “yes” for every trial). The visualizations varied across the studies: in Study 1–3, we used scatterplots built with the d3 toolkit,^65^ with a white background and one-pixel gray axes without labels or numbers (the presence of axes is in contrast to classic visual search experiments).^3,5,14,66^ The visualizations were displayed in a web browser as shown in Figure 1.

Scatterplots were made up of *targets* (i.e. emphasized objects) and *distractors* (i.e. data items that were not emphasized). We ensured that target items were never overlapped by distractor items. To increase the ecological validity of our stimuli, we approximated real-world scatterplots by adding $\sqrt{\frac{\mathrm{visualization}\;\mathrm{area}}{\mathrm{mark}\;\mathrm{diamete}r^{2}}}$ distractor marks at random positions. This approach is specifically intended to avoid the Isolation Assumption that is used in previous color science studies and models (i.e. that viewers are making comparisons between two isolated stimuli^67^), which is unrealistic in a typical visualization setting where visualization designers cannot dictate the number or location of items. Since targets were a random distance from distractors in each trial, this approach also avoids an effect seen in previous research in which the distance between distractor and target can influence color difference perceptions.^68^

### Stimuli: Twelve emphasis effects

**Figure 2.**
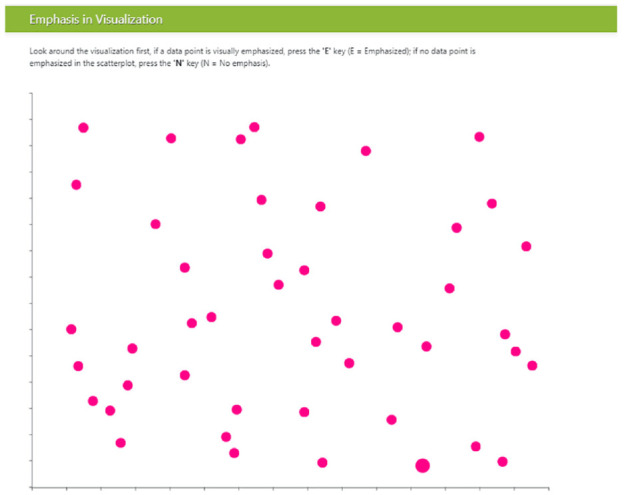
Example distractors (*D*), stimuli, and their magnitude ranges.

We chose visual variables that have been shown in previous research to pop out in a pre-attentive manner, that have been evaluated in previous visualization work^3,4,20,48^ and that are available in commercial tools such as Tableau and visualization libraries such as D3.js,^65^ VegaLite,^69^ Plotly, and ggplot2. For all visual variables except shape, we created six levels of the effect to examine increasing difference between the target and the distractors – we call this “magnitude of difference.” Six levels were chosen to give us a wide range for each effect, while accounting for some effects that can be limited at either end of the scale (such as opacity), while others may not have such limits (such as size or motion). These ranges expand those seen in previous work^4,48^ and allow us to model each effect and enable comparisons between them. The levels of each variable are described below and illustrated in Figure 2.

#### Time-invariant effects

Color: We chose six magnitude levels from the perceptually uniform CIE LAB color space. In this space, the Euclidean distance between a pair of colors determines their perceptual distance: if two pairs of colors have the same Euclidean distance between the members of the pair, then their perceptual distance is the same. To accurately compare a range of colors we use the current ΔE standard, CIEDE2000^70,71^ as our primary color difference metric. Our six color differences (i.e. the difference between emphasized and non-emphasized elements) ranged from ΔE 5 to ΔE 30.

*Size*: Previous research has determined that perceived size can be different from geometric size, so we included this adjustment in the calculation of the size difference for each magnitude level. We used the equation *Perceived size* = *Actual size*^0.86^, where actual size signifies the area of the object, as suggested by previous work.^72^ For consistency with prior work,^48^ we chose six sizes for the target, with each level increasing the target’s difference compared to the distractors by 0.25x in perceptual space; therefore, the target’s size ranged from 1.25x at level one up to 2.5x at level six.

*Blur/Focus*: Blur/Focus is applied to the non-emphasized points of the visualization, so that the target remains sharp while the distractors are blurred. We chose six different blur intensities – with blur radius ranging from 0.5 to 3.0 pixels. We used Javascript’s <feGaussianBlur> SVG filter for our blur effect.

*Opacity*: Similar to Blur/Focus, opacity effects are applied to non-emphasized items, such that the target remains fully opaque while distractors become more transparent. Opacity is limited in range at both endpoints, with a maximum opacity of 1 and a minimum of 0.1; thus, we chose our range to be within those bounds. Opacity on the distractors was varied from 0.85 (nearly opaque) to 0.1 (nearly transparent).

*Shape*: Although shape is commonly used in visualizations, this visual variable is typically used for categorical rather than linear data. Previous research has not considered whether arbitrary shapes have differences in noticeability – that is, whether there is an inherent ordering of shapes that could be used by designers to add emphasis effects (although see discussion of QTON shapes below). To test whether shapes differ in how they convey emphasis, we chose three simple shapes that are available in common visualization tools such as D3 and Tableau: square, star, and diamond (in addition to the circular distractors). We tested the shapes in both filled and unfilled styles, giving six shapes in total (distractors had the same fill as the target – e.g. if the target was filled then the distractors were also filled). Because we did not assume any a priori ordering, Shape is not included in our magnitude analyses below. The shapes we used are illustrated in Figure 2.

**Figure 3.**
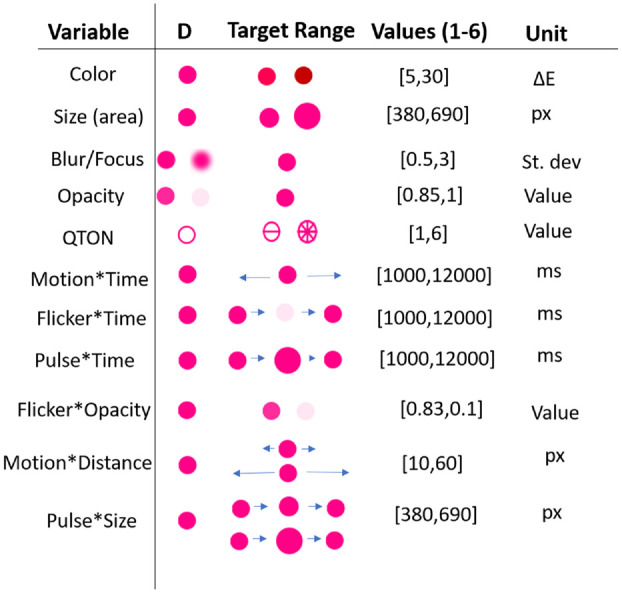
Shapes and QTONS used in our studies.

*QTONS*: Ware’s^73^ Quantitative Texton Sequences (QTONS) are a set of shape glyphs that previous research claims can be perceptually ordered – that is, such that each glyph in the sequence is visually distinct and is perceived as increasing or decreasing in value. The QTONS we used are illustrated in Figure 3: the series adds lines to a base circular representation to increase perceptual distance from a plain circle.

#### Time-variant effects

We also developed two groups of emphasis effects that use time – these combine one of the visual variables described above with a change over time. As described below, the two groups differ in how they create different magnitude levels: the first three effects hold the two endpoint values of the visual variable constant, and change the amount of time over which the value varies; the second group of effects holds the time constant, and varies the endpoints of the visual variable.

Pulse*Time: Pulse is the variation of size over time. The magnitude level controls the amount of time taken for a transition from smallest size to largest size; we used six time durations from 1000 to 12,000 ms for our magnitude levels. Size changed from 1.0x (the size of the distractor) to 2.5x (the maximum used for the Size variable described above).

Flicker*Time: Flicker uses the Opacity emphasis effect described above, but varies opacity over time. We used six time durations from 1000 to 12,000 ms as our magnitude levels, with opacity changing from 0.0 (completely opaque) to 1.0 (completely transparent).

Motion*Time: Motion is variation of position over time. We used six time durations from 1000 to 12,000 ms as our magnitude levels. Targets moved 50 pixels left from their starting position, then 50 pixels right, then back to the starting position.

Pulse*Size: We created a second variation of our pulse effect, where instead of controlling the speed of the transition, the magnitude level controlled the amount of change in size over a fixed time interval (500 ms). For this effect, targets changed from 1.0x to the six sizes described above for the Size effect.

Flicker*Opacity: Our second variation for Flicker used different changes in opacity as our magnitude levels, over a fixed time interval (500 ms). For this effect, targets changed from completely opaque to one of six equally-spaced opacity levels between completely opaque and completely transparent.

Motion*Distance: Our second variation for motion controlled the amount of displacement of the target stimulus, while keeping the time interval fixed (500 ms). For this effect, targets moved an amount equal to the magnitude level*10 pixels ( distance=magnitude*10 ). Target movement followed the same left-then-right path described above.

### Procedure for all studies

Each experiment followed six phases: (1) informed consent, (2) demographics questionnaire, (3) vision test, (4) tutorial, (5) study tasks, and (6) debriefing. The specific questions and tasks for each study are described in each experiment’s section below. Participants first completed informed consent and demographics forms, and were then asked to complete an Ishihara^74^ test and questionnaire to screen for color vision deficiencies. Participants then completed a set of tutorial examples, where they had to answer all questions correctly to proceed with the study.

Participants then completed the study tasks. Ordering of trials presented stimuli in random order, to account for attention theories that suggest visual attention can be guided by previously seen targets^15^ and to prevent learning effects. In addition, while some visual search studies show participants a pre-defined target before a trial,^4,11^ we opted not to present the target beforehand because of the *relational account of attention* theory, which suggests that viewer’s attention in tasks without a specific target will be guided toward the mark that differs the most from all other marks in a given visual variable^75^; this represents a better measure for perceived emphasis. All participants completed all trials of each study. We followed standard signal-detection methodology for recording participant responses^42^: participants pressed “E” to indicate that they saw an emphasized item, and “N” to indicate that there was no emphasized item. In trials where participants responded that an emphasis effect was present, a pop-up box appeared, asking participants to rate how different the emphasized point was from the other points (e.g. if it was a different color, how different), on a 1–7 scale where 1 = “slightly different,” and 7 = “very different.” After each trial and response, the screen was blanked for 0.5 s to prevent contrast effects between trials. After completing all trials, participants were then shown a debriefing form and were compensated for their participation.

To help us identify potential negligent workers (e.g. those who answer “yes” to every trial), filter out online bots,^76^ and ensure data quality, we included two primary engagement checks in accordance to our planned analysis. First, we expected trials with no emphasis effects to have a high accuracy (we determined a minimum 70% accuracy). Similarly, we expected trials that have a high magnitude level (e.g. a magnitude of 5 or above) to also have a minimum 70% accuracy for specific variables such as motion or any shape stimuli. We identified these thresholds with preliminary data gathered from our early testing of the experimental software. Data from participants who failed a majority of these engagement checks was not included in the analysis, but they were still compensated upon completion of the study.

### Participant recruitment

We recruited 200 participants across four experiments using Amazon’s Mechanical Turk (MTurk), and gathered data with a custom browser-based experiment tool.^77^ MTurk is an online platform where requesters can post tasks that workers can opt-in to complete. Data collected from MTurk has been previously used to model perception in visualization,^41,64^ including assessing separability of variables,^29^ measuring colormaps,^63^ and effectively detecting motion,^24^ as well as in a variety of human-computer interaction studies.^78–81^

While removing participants on the basis of an engagement check can potentially introduce post-treatment bias,^82^ care must be taken to ensure data quality by filtering bots or negligent workers from online crowdsourcing platforms.^41,76,83–86^ For all experiments, we restricted the participants to be within the United States and to have an acceptance rate (a measure of a worker’s past quality) over 90%.

### Analysis and pre-registration

The main goal of the studies was to gather empirical data about the perceptibility of emphasis effects under different conditions. To investigate these differences, each study used a repeated-measures design with two main factors:

- Emphasis effect
- Magnitude of difference

Our analysis for Magnitude investigates how the perception of each visual variable is affected by increasing its strength. Additional factors (e.g. number of distractors, visualization complexity, and visualization type) will be described in each study’s section below.

We used three dependent measures that provide us with insights about the user’s experience with emphasis: *search time* (the time between stimulus onset and the user’s keypress) provides an indication of pre-attentive visual attention; *correctness* of the response evaluates the user’s conscious decision about the visibility of the emphasis effect; and *visual rating* provides us with details about how obvious the effect was to the user. Spatial locations of target elements was random. We report effect sizes for significant RM-ANOVA results as general eta-squared $\eta^{2}$ (considering 0.01 small, 0.06 medium, and >0.14 large^87^). For all follow up tests involving multiple comparisons, the Holm correction was used.

Cockburn et al.^88,89^ recently reviewed the many benefits of pre-registering research hypotheses, study methods, and data analysis plans. We pre-registered our data analysis approach using the persistent data storage of the Open Science Framework (OSF) (https://osf.io/5pfh4?view_only=ec9a5ec5618b4b348ae3cb990f45c402).

## Experiment 1: Establishing a baseline of perceived emphasis

The first experiment focuses on participants’ abilities to accurately detect when an emphasis effect is present in a visualization, allowing us to explore the relative differences between effects, determine a baseline for perceived emphasis, and build an initial predictive model for basic scatterplot visualizations. Each participant completed 184 trials: two trials per *emphasis effect*×*magnitude* combination (144 total), and 40 trials with no stimulus present (as a manipulation check). The study procedure took approximately 30 min to complete, and participants were compensated with $7 USD for their participation.

### E1 results

We recruited 50 participants for this experiment from Amazon’s Mechanical Turk platform (participants who did not complete the study were replaced). From the 50 successful completions, we excluded five participants from analysis following our pre-registered criteria for either failing to correctly answer a majority of the engagement checks (including skipping trials or rating questions (four) or having an overall completion time outside 3 SD (one) from the mean). We additionally removed eight trials that had a completion time greater than 3 SD from the block mean. We analyzed data from the remaining 45 participants ( $\mu_{\mathrm{age}}$ = 39.68, $\sigma_{\mathrm{age}}$ = 11.74, 29 men, 16 women). Summary charts including removed participants are available in the Supplemental Appendix.

**Figure 4.**
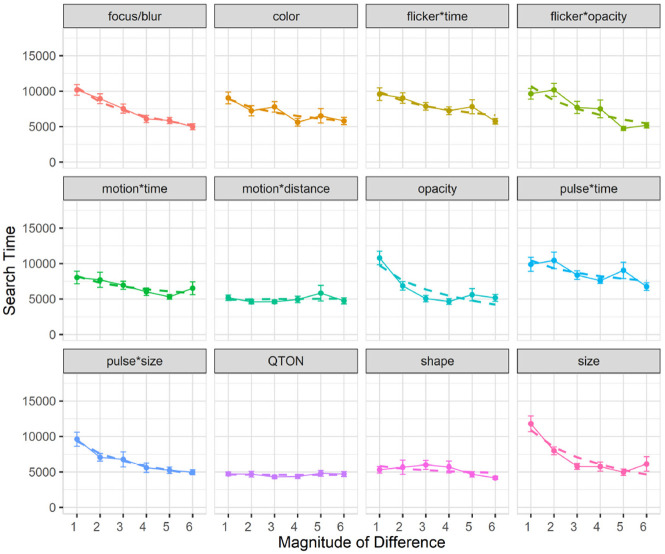
Experiment 1, mean trial time (±SE) per variable. Empirical means (solid lines) and log curve (dashed lines).

Search time: Search time was measured as the time between stimulus onset and the user’s keypress. A 12×6 RM-ANOVA showed significant main effects of both *Emphasis Effect* ( $F_{11,484}=16.04,p<0.001,\eta^{2}=0.06$ ) and *Magnitude of Difference* ( $F_{5,220}=25.93,p<0.001,\eta^{2}=0.06$ ) on search time, and an interaction between the factors ( $F_{55,2420}=1.52,p<0.001,\eta^{2}=0.03$ ). The data are summarized in Figure 4.

Across all magnitudes, search time was lowest with the QTON effect (mean 4599 ms) and motion*distance (4990 ms); the pulse*time effect had the largest search times (8685ms). Across all effects, search times consistently decreased as magnitude of the effect increased – from 8654 ms at level 1 to 5410 ms at level 6 (see Figure 4).

However, two effects do not follow this decreasing trend, leading to the interaction between the two main factors. The perceptibility of the motion*distance effect and the QTON shapes both change very little as magnitude increases, suggesting that there is little perceptual difference across different motion distances or across the different QTON shapes (even though these were designed to be perceptually ordered).

We carried out follow-up one-way analyses to further explore the main effects. The follow-up tests indicated that emphasis effects can be grouped based on perceptual commonalities. Some of these groups were expected: for example, all time-variant effects had similar performance (all *p* > 0.05 for pairwise comparisons). Other groupings were not expected: for example, the focus/blur time-invariant effect was similar to time variant effects such as pulse*size, flicker*opacity, and motion (all *p* = 1.0).

Follow-up analysis of effect magnitude showed significant differences between level 1 and levels 2–6, and between level 2 and levels 4–6 (all *p* < 0.05).

**Figure 5.**
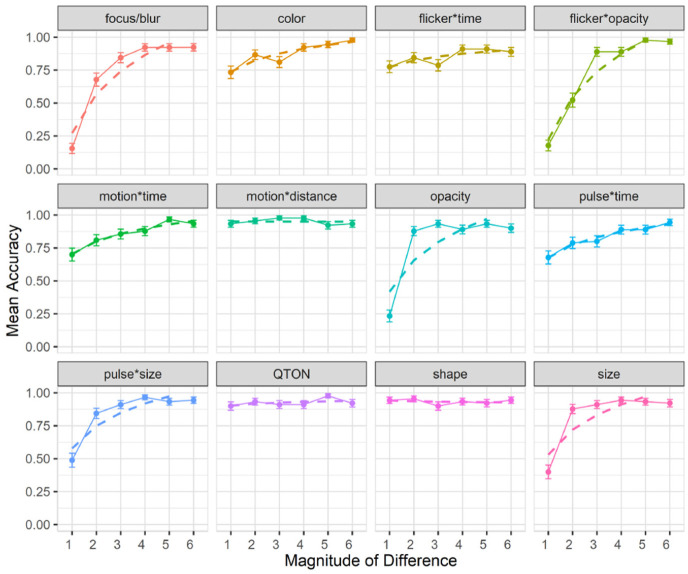
Experiment 1, mean accuracy (±SE) per variable. Empirical means (solid lines) and log curve (dashed lines).

Accuracy: Accuracy was measured as the fraction of trials where an emphasized target was correctly detected. A 12×6 RM-ANOVA showed significant main effects of both *Emphasis Effect* ( $F_{10,440}=10.62,p<0.001,\eta^{2}=0.07$ ) and *Magnitude of Difference* ( $F_{5,220}=128.41,p<0.001,\eta^{2}=0.21$ ) on accuracy, as well as an interaction between the factors ( $F_{55,2200}=16.96,p<0.001,\eta^{2}=0.18$ ). The results are summarized in Figure 5.

The charts show that some effects such as focus/blur, opacity, flicker*opacity, size, and pulse*size are substantially harder to perceive at lower magnitude levels; other effects such as color, flicker*time, motion*time, pulse*time, and pulse*size have a gradual improvement with increasing magnitude; and a third group of effects such as motion*distance, QTONs, and Shape have a near-100% detection rate regardless of magnitude. These differences explain the interaction between the two main factors (the interpretation of these interactions is considered further in the Discussion section below).

Across all magnitudes, participants had highest accuracy with motion*distance at 0.95, while focus/blur had the lowest detection rate at 0.74. Across all effects, detection accuracy changed from 0.59 at level 1 to 0.93 at level 6. In addition, across all effects we found a False-Positive rate of 10% and a False-Negative rate of 15%.

Follow-up tests showed similar groupings to those found in our analysis of search time. Focus/blur was again found to be perceptually similar to time-variant effects such as pulse*time (*p* = 1.0), and Shape, QTONs, and motion*distance formed a group with similar detection rates (all *p* = 1.0).

**Figure 6.**
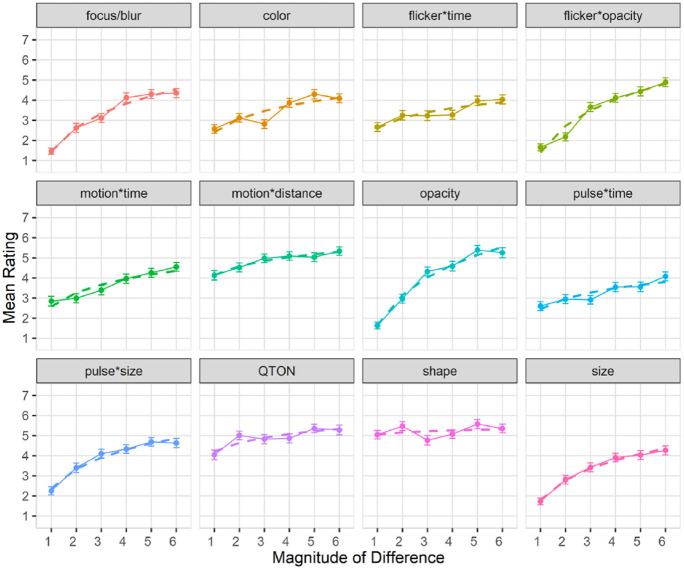
Experiment 1, mean rating (±SE) per variable. Empirical means (solid lines) and log curve (dashed lines).

Subjective perception of magnitude of differences: After the presentation of each visualization, participants were asked to rate how different the emphasized point was from the other points on a 1–7 scale. Mean response scores are shown in Figure 6. We used the Aligned Rank Transform^90^ with the ARTool package in R to enable analysis of the subjective prominence responses. RM-ANOVA showed significant effects for *Emphasis Effect*, *Magnitude of Difference*, and an interaction between the factors (all *p* < 0.001). Overall, subjective results follow those of accuracy, with participants rating the perceptibility of shapes and QTONs as high, regardless of magnitude (although unlike the objective data, participants did rate QTONs higher as magnitude increased). Subjective ratings ranged from lows of under 2 (on a 7-point scale) at magnitude level 1 (e.g. focus/blur, opacity, flicker*opacity, and size) to highs above 5 at magnitude level 6 (e.g. opacity, motion*distance, QTONs, and Shape).

### Exploratory analyses

The analyses described above provide insights on the main effects of variable and magnitude on perceptibility. To provide deeper insights on the performance of the different emphasis effects, we carried out a series of follow-up analyses on different aspects of the effects. As we did not pre-register these analyses, we present the results as preliminary and as requiring a planned analysis to be confirmed in future work.

First, our emphasis effects can be classified into two groups: time-variant and time-invariant effects, with six effects in each group. We carried out 1×2 RM-ANOVAs to look for differences between these groups. For search time, the analysis showed a significant main effect of *Type of Effect* (time-variant vs time-invariant) ( $F_{1,44}=28.80,p<0.001,\eta^{2}=0.003$ ) on search time, with time-invariant effects having a shorter search time (6257 ms) than time-variant effects (7058 ms). For accuracy, we found no effect of *Type of Effect* on accuracy ( $F_{1,44}=0.73,p=0.3$ ).

Second, our time-variant effects can be subdivided into two main groups depending on how the time variation is achieved. As described in the Stimuli section above, effects can either hold the two endpoint values of the visual variable constant, and change the duration of the effect; or the effect can hold duration constant, and vary the endpoints of the visual variable. For search time, a 1×2 RM-ANOVA showed a significant main effect of *Type of Time-Variance* ( $F_{1,44}=31.9,p<0.001,\eta^{2}=0.009$ ) on search time, with constant-duration effects (pulse*size, motion*distance, and flicker*opacity) having lower search times (6344 ms) than variable-duration effects such as pulse*time, motion*time, and flicker*time (7774 ms). For accuracy, a 1×2 RM-ANOVA found no effect of *Type of Time-Variance* on accuracy ( $F_{1,44}=0.027,p<0.87$ ).

Third, we also examined differences between filled and unfilled shapes (see Figure 3) within the Shape effect. A 1×2 RM-ANOVA found no effect of Shape Fill on search time ( $F_{1,44}=2.392,p=0.12$ ) or accuracy ( $F_{1,44}=0.001,p=0.97$ ).

### Modeling perceptibility

Following a similar method described in previous work^48^ we used the raw data from Study 1 to build predictive models of emphasis perceptibility based on magnitude of difference, for trial times, accuracy, and subjective rating. We used logarithmic functions as our model, as these are commonly used to describe human performance in signal-detection and perceptual studies.^91^ The logarithmic fit functions can be seen as the dashed lines in Figures 4 to 6. We fit the functions to the data using R (lm(mean ~ log(magnitude of difference)); this log model offers a relatively good fit for most variables, with $R^{2}$ as high as 0.93 for pulse*time’s accuracy and average $R^{2}$ value of 0.72 (0.21 SD). We then use R’s “predict” function to get predicted values which will be used to compare performance for emphasis effects across visualization types in the fourth experiment.

## Experiment 2: Effect of number of distractors on emphasis

Our first experiment showed that emphasis effects differ in terms of perceptibility, and that there are differences in perceptibility with increasing magnitude. The first study used scatterplots where the density of distractors was relatively low, and because the amount of visual information in a visualization – also called *clutter*– can vary widely, our second study investigates how an increasing number of distractors in the display affects emphasis perceptibility.

Previous research suggests that elements that “pop out” can be processed rapidly and independently of the number of distractors.^3–5^ While there are many ways clutter can be described and quantified within a visual display (see Rosenholtz et al.^92^ for a review), for this evaluation we opt for a simple metric of “fraction of the display area covered by distractors”– a metric that is applicable for designers who cannot control how the data will look (e.g. data point quantities or their distributions on a screen).

### Stimuli

We used the same experimental setup as in study 1, but with a subset of magnitude levels (1, 3, and 5) for each variable, which for the shape variable corresponded to the filled square, filled diamond, and unfilled star. We created a new factor called *clutterLevel* to control the number of background distractors. Level 1 represents a baseline, and is the same amount used in study 1 (where distractors cover approximately 2% of the available visualization space). ClutterLevel 2 increases distractor coverage to approximately 40%, while clutterLevel 3 represents 80% coverage.

### Experimental design

Through the same binary forced-choice task as study 1, we measured perceived emphasis across different magnitude levels and clutter levels. Again, we used search time, accuracy, and subjective rating of difference between the emphasized element and the distractors as our primary dependent measures. Each participant completed 153 trials: one trial per combination of *emphasis effect*×*magnitude*×*clutterLevel* (108 total), and 45 trials with no stimulus present (15 trials per clutter level).

### E2 results

We recruited 50 new participants, and replaced anyone who did not complete the study. Using our 50 successful completions, we followed a similar exclusion criteria as in Study 1; two participants were excluded from our analysis for failing a majority of engagement checks (including skipping trials and rating questions). We analyzed data from the remaining 48 participants ( $\mu_{\mathrm{age}}$ = 36.56, $\sigma_{\mathrm{age}}$ = 10.46, 30 men, 17 women, 1 no response). Thirteen trials were identified as outliers (completion time above 3 SD from the block mean) and were excluded from our analysis. Our analysis considers emphasis effect, magnitude, and clutter level in order to look for main effects of clutter on search times, accuracy, and subjective ratings, and to look for interactions between clutter and the other factors. As with Study 1, we report effect sizes as general eta-squared $\eta^{2}$ , and use Holm correction for follow-up tests.

#### Effects of clutter on search time

**Figure 7.**
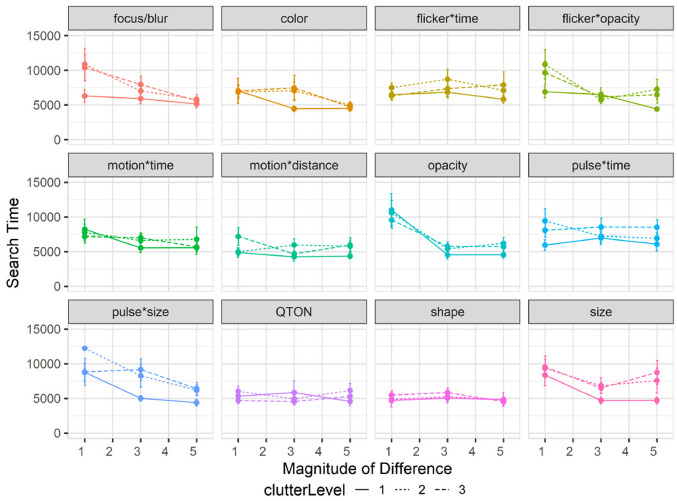
Experiment 2, mean search times (±SE) per variable with multiple distractor amounts. Level 1 (solid lines), levels 2–3 (dashed lines).

Overall search time was slightly faster for Study 2 at 6619 ms compared to 6872 ms from study 1. A 12×3×3 RM-ANOVA (*Emphasis Effect*×*Magnitude of Difference*×*ClutterLevel*) showed a significant main effect of Clutter Level on search time ( $F_{2,94}=14.98,p<0.001,\eta^{2}=0.006$ ), and also showed effects of Emphasis Effect ( $F_{10,470}=4.91,p<0.001,\eta^{2}=0.01$ ) and Magnitude of Difference ( $F_{2,94}=29.180,p<0.001,\eta^{2}=0.01$ ). As shown in Figure 7, search times were lowest with clutter level 1, with increases for clutter levels 2 and 3. Post hoc *t*-tests showed significant ( p<0.001 ) differences between *ClutterLevel* 1 → 2 and 1 → 3, but not between 2 → 3 (*p* = 0.42).

The RM-ANOVA did not show any significant interactions between Clutter Level and either Emphasis Effect ( $F_{20,940}=1.02,p=0.44$ ) or Magnitude ( $F_{4,188}=0.73,p=0.39$ ). The lack of interaction between Clutter Level and Emphasis Effect indicates that most effects saw consistent changes based on numbers of distractors. Similarly, there was no interaction between Clutter Level and Magnitude. Although the interactions were non-significant, it may be valuable to look further at these combinations in future studies.

In particular, emphasis effects involving size appeared to be more affected by clutter than other visual variables. While it has long been believed that size and other variables such as color are separable^93^– that is, that either of these visual variables can be attended to without interference from the other – recent work challenges this notion.^29,58,67^ As such, varying the number and location of distractors may affect size perceptions. While we controlled the layout of distractors so that there would be no overlaps, it is possible that increasing number of distractor made differences in size more difficult to perceive for our participants.

#### Effects of clutter on accuracy

**Figure 8:**
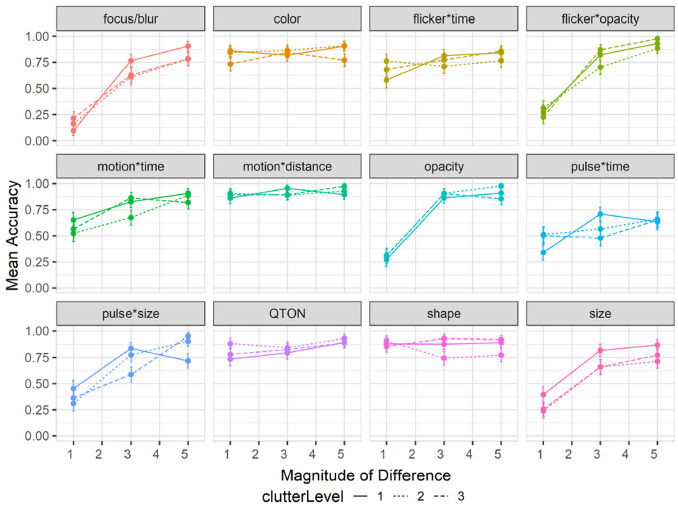
Experiment 2, mean accuracy (±SE) per variable with multiple distractor amounts. Level 1 (solid lines), levels 2–3 (dashed lines). .

Results are summarized in Figure 8. Overall accuracy in study 2 went down to 78% compared to study 1 (85%). A 12×3×3 RM-ANOVA did not show a significant main effect of *Clutter Level* on accuracy ( $F_{2,94}=0.4,p=0.1$ ), but did find main effects of *Emphasis Effect* ( $F_{10,470}=32.33,p<0.001,\eta^{2}=0.06$ ) and *Magnitude of Difference* ( $F_{2,94}=83.20,p<0.001,\eta^{2}=0.09$ ). There were no significant interactions between *Clutter Level* and *Emphasis Effect* ( $F_{20,940}=1.09,p=0.43$ ) or *Clutter Level* and *Magnitude* ( $F_{4,188}=0.07,p=0.93$ ). While clutter was not found to significantly affect the accuracy at perceiving an emphasized target, we must note that increasing number of distractors increased the overall False-Positive rate to 19% and False-Negatives to 27%, from 10% and 15% from study 1 respectively.

#### Effects of clutter on perceived difference

**Figure 9.**
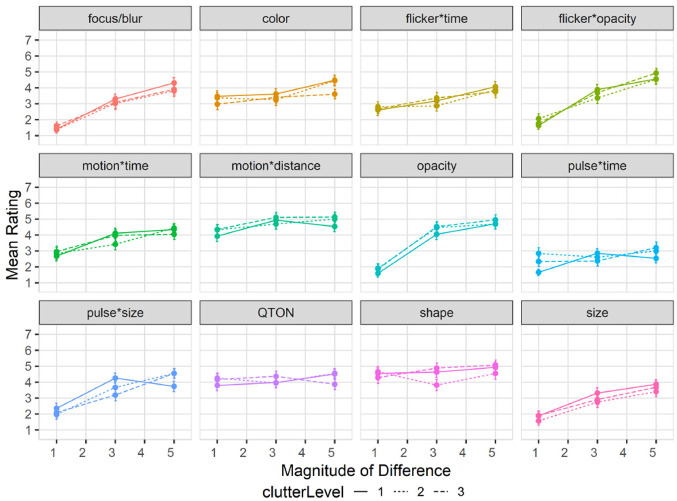
Experiment 2, mean rating (±SE) per variable with multiple distractor amounts. Level 1 (solid lines), levels 2–3 (dashed lines).

Mean rating response scores are shown in Figure 9. We again used the Aligned Rank Transform to enable ANOVA on subjective ratings. We again found main effects (all *p* < 0.01) of *Emphasis Effect* and *Magnitude of Difference* on subjective rating, but no main effect of *Clutter Level* (*p* = 0.22). The rating scores in this study largely matched those of study 1, following the same trend of increasing rating as magnitude increased for most variables, and the visual rating of all variables remained similar regardless of distractor amounts.

### Exploratory analyses

Similar to Study 1, we also carried out follow-up analyses to explore clutter’s effect on different groupings within the emphasis effects. First, we again considered time-variant versus time-invariant emphasis effects. For search time, a 2×3 RM-ANOVA (*Type of Effect*×*Clutter Level*) showed no interaction between the factors ( $F_{1,47}=0.48,p=0.48$ ), indicating that the effects of clutter were similar across both types of effects. For accuracy, a similar RM-ANOVA also showed no interaction ( $F_{1,47}=0.56,p=0.46$ ). Other results from the RM-ANOVA mirror those of Study 1: a main effect of *Type of Effect* on search time ( $F_{1,47}=7.34,p=0.006,\eta^{2}=0.002$ ) with time-invariant effects faster (mean 6324 ms) than time-variant (mean 6913 ms), and no main effect of *Type of Effect* on accuracy ( $F_{1,47}=0.23,p=0.6$ ).

Second, we considered the different types of time variance, and again subdivided our time-variant effects into two groups (constant-duration or variable-duration). For search time, a 2×3 RM-ANOVA (*Type of Time-Variance*×*Clutter Level*) found an interaction between the factors ( $F_{1,47}=5.89,p=0.02$ ). For accuracy, a similar RM-ANOVA showed no interaction ( $F_{1,47}=0.04,p=0.6$ ). In contrast to Study 1, the RM-ANOVA showed no main effect of *Type of Time-Variance* ( $F_{1,47}=1.78,p=0.18$ ) on search time, but did show an effect on accuracy ( $F_{1,47}=8.75,p=0.003,\eta^{2}=0.003$ ), with constant-time effects (e.g. pulse*size, motion*pixels, and flicker*opacity) having a higher accuracy (74%) than variable-time effects (68%).

## Experiment 3: Effects of mixed distractor types on perception of emphasis

Our first two studies both used emphasis effects that were strongly different from the visual representation of the distractors – that is, both studies harness the “pop out” phenomenon, where a target defined by a unique visual feature can be pre-attentively detected^3,5^ regardless of the number of distractors (although with some exceptions as shown by study 2).

However, it is known that mixing distractors to create a “feature conjunction” task makes visual search more difficult^3,5,11^ and can reduce the pop-out effect, as search efficiency is guided by the difference between the distractors.^11^ Therefore, our third study evaluates perceptibility of emphasis in a feature conjunction task where distractors are mixed, using a similar task paradigm to that outlined in the General Methods section.

### Stimuli

**Figure 10.**
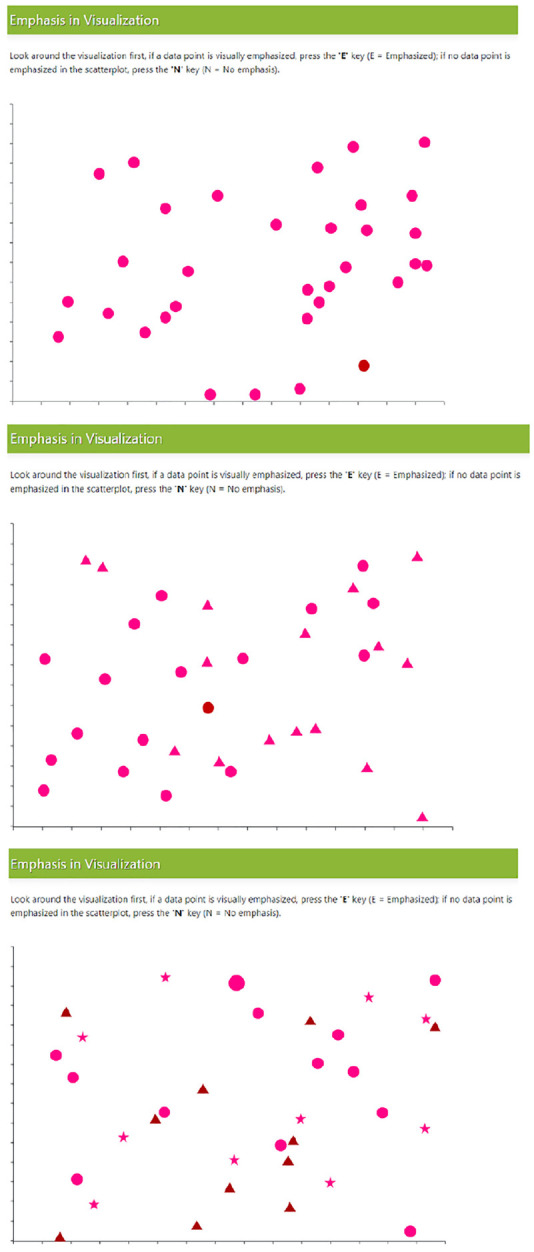
Example trials with multiple distractor types. Top (one distractor), middle (two distractors), and bottom (three distractors).

We again used a subset of Study 1’s magnitude levels for Study 3 – we chose three uniform steps (1, 3, and 5) from Study 1, giving us coverage of the range we used to establish our baseline results. For Study 3, we add a new factor –*distractor type count*– that indicates the number of distractor types in the task. We used shape as the primary visual variable manipulated by the factor, and added three levels (1, 2, and 3 distractor types): level 1 has only one distractor type (a filled circle), and so is similar to conditions seen in Study 1; level 2 has two distractor types (a filled triangle in addition to the filled circle) where each type is used for 50% of the distractors; level 3 has three types (a filled star, a filled triangle, and the filled circle) with each type used for 33.33% of the distractors. In level 3, we additionally manipulated the filled triangle’s color on trials that did not involve using color difference to emphasize a target to further increase the difference between distractors, as it is known that increasing distractor heterogeneity decreases search performance.^11^ Examples of each level are depicted in Figure 10.

### Experimental design

Our experiment again used a binary forced-choice task measuring perceived emphasis difference across different magnitude levels and different numbers of distractor types. We used accuracy, search time, and visual rating as our primary dependent measures for this study. Each participant completed 153 trials: one trial per combination of *emphasis effect*×*magnitude*×*distractor type count* (108 total), and 45 trials with no stimulus present (15 trials per distractor type count).

### E3 results

We recruited 50 new participants for this experiment (replacing any who did not complete). From the 50 successful completions, two people were excluded from our analysis following a similar exclusion criteria from studies 1 and 2, such as failing engagement checks, including skipping trials or rating questions. The study procedure took approximately 30 min to complete, and participants were compensated with $7 USD for their participation. We removed 25 trials from our analysis as outliers based on having a completion time greater than 3 SD from the block mean. We analyzed data from the remaining 48 participants ( $\mu_{\mathrm{age}}$ = 36.63, $\sigma_{\mathrm{age}}$ = 10.47, 28 men, 18 women, 2 DNR). We analyzed distractor type count, emphasis effect, and magnitude of difference on our three dependent measures. As in our first two studies, we report effect sizes as general eta-squared $\eta^{2}$ , and use Holm correction for follow-up tests.

**Figure 11.**
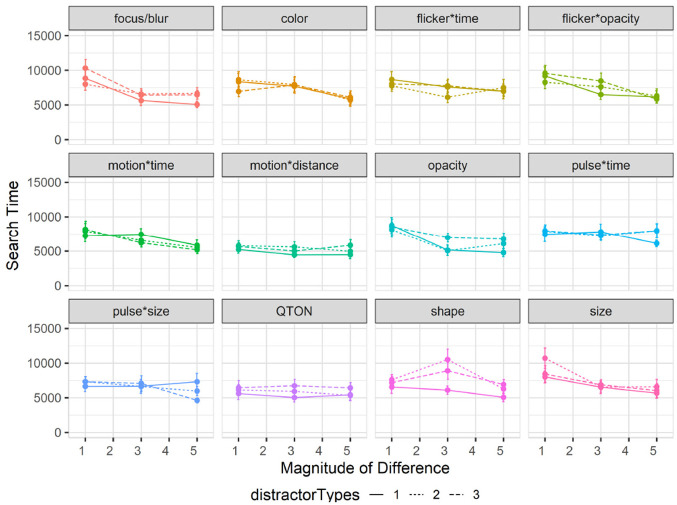
Experiment 3, mean search times (±SE) per variable with multiple distractor types. Level 1 (solid lines), levels 2–3 (dashed lines).

Effects of distractor types on search time: Overall search time in study 3 increased to 7149 ms compared to 6872 ms in study 1. A 12×3×3 RM-ANOVA (*Emphasis Effect*×*Magnitude of Difference*×*Distractor Types*) showed no main effect of *Distractor Type Count* on search time ( $F_{2,94}=1.48,p=0.14$ ), but there was an interaction between *Emphasis Effect* and *Distractor Type Count* ( $F_{22,968}=4.0,p<0.001,\eta^{2}=0.03$ ). As can be seen in Figure 11, the interaction is largely driven by the Shape emphasis effect, which had higher search times with two types of distractors (overall mean of 7880 ms) and three types (7612 ms) than with only one type of distractor (5756 ms). None of the other emphasis effects saw clear differences in search time as the number of distractor types increased.

The other results of the RM-ANOVA largely follow those of Study 1: there were again main effects of *Emphasis Effect* ( $F_{11,517}=6.22,p<0.001,\eta^{2}=0.01$ ) and *Magnitude of Difference* ( $F_{2,94}=32.92,p<0.001,\eta^{2}=0.01$ ) on search time.

Effects of distractor types on accuracy: Overall accuracy in study 3 also decreased to 78% compared to 85% in study 1. A 12×3×3 RM-ANOVA showed a significant main effect of *Distractor Type Count* on accuracy ( $F_{2,94}=28.60,p<0.001,\eta^{2}=0.01$ ), and also showed effects of *Emphasis Effect* ( $F_{11,517}=25.47,p<0.001,\eta^{2}=0.04$ ) and *Magnitude of Difference* ( $F_{2,94}=32.92,p<0.001,\eta^{2}=0.08$ ). A pairwise test between distractor type count showed significant differences between levels 1 and 3 (*p* < 0.001) and 2 and 3 (*p* = 0.014). The main effects of Emphasis Effect, Magnitude, and Distractor Type Count on accuracy must be considered in light of the interactions described below.

**Figure 12.**
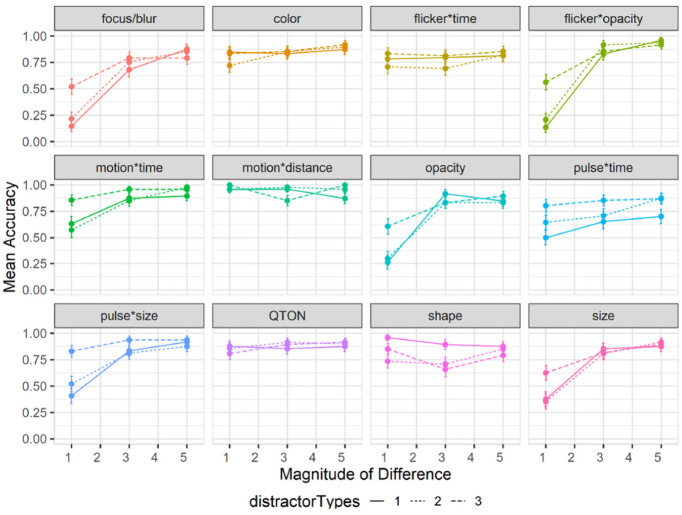
Experiment 3, mean accuracy (±SE) per variable with multiple distractor types. Level 1 (solid lines), levels 2–3 (dashed lines).

Interactions between emphasis effect, magnitude, and distractor type count: RM-ANOVA showed significant two-way interactions between *Emphasis Effect* and *Distractor Type Count* ( $F_{22,1034}=4.01,p<0.001,\eta^{2}=0.014$ ) and *Magnitude of Difference* and *Distractor Type Count* ( $F_{4,188}=16.49,p<0.001,\eta^{2}=0.08$ ). These interactions are illustrated in Figure 12.

The added factor of Distractor Type Count made certain effects easier to notice particularly at magnitude level 1 (e.g. pulse*size had an accuracy of 0.48 with one distractor type compared to 0.82 with all three distractor types, and focus/blur had an accuracy of 0.14 at magnitude 1 with one distractor type compared to 0.52 with three distractor types). In general, we also saw no effect of distractor type count on accuracy for Shape; however, this could have been guided by our choice of tested shapes, as we saw a drop in accuracy for the filled diamond shape from 93% with one distractor type to 69% with two types and 78% with three types. Duncan and Humphrey’s^11^ study and a more recent study by Haroz and Whitney^31^ found very small performance costs of a few milliseconds per additional distractor type or category for visual search tasks. Our results show that this minimal performance cost remains true for most emphasis cues, with some considerations needed for size-based effects.

The additional distractor types increased search difficulty in trials with no target present (i.e. increasing completion time and lowering accuracy). Similar to Study 2, and compared to the baseline Study 1, we note that increasing distractor types increased the overall False-Positive rate to 29% and False-Negatives to 22%, from 10% and 15% in Study 1.

**Figure 13.**
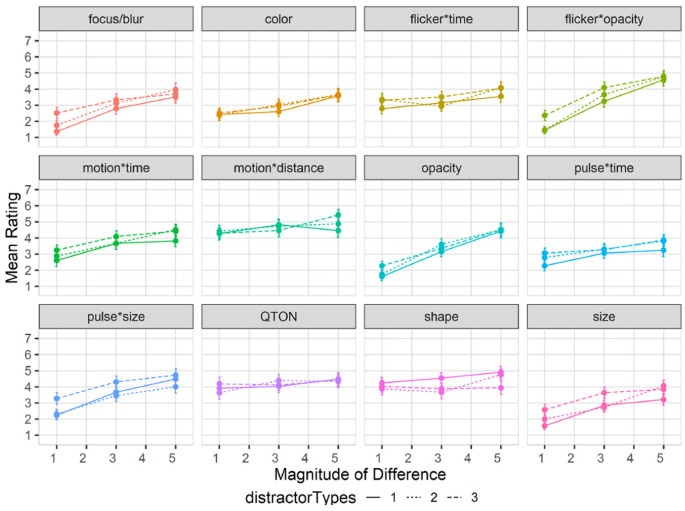
Experiment 3, mean rating (±SE) per variable with multiple distractor types. Level 1 (solid lines), levels 2–3 (dashed lines).

Effects of distractor types on subjective perception of magnitude of difference: Mean rating response scores are shown in Figure 13. We carried out an ANOVA using the Aligned Rank Transform on subjective ratings of perceived emphasis. There were main effects (all *p* < 0.01) of *Emphasis Effect* and *Magnitude of Difference*, but not *Distractor Type Count* (*p* = 0.18). The rating scores in this study largely matched those of study 1, following the same trend of increasing rating as magnitude increases for most variables, and the perception of all variables remained similar regardless of distractor count.

### Exploratory analyses

We carried out follow-up analyses to investigate effects of distractor type on the different groups of emphasis effects. First, we considered time-variant versus time-invariant effects. For search time, a 2×3 RM-ANOVA (*Type of Effect*×*Number of Distractor Types*) found no main effect of Type of Effect ( $F_{1,47}=0.12,p=0.73$ ) and/did not find a significant interaction between the factors ( $F_{2,94}=0.69,p=0.54$ ). For accuracy, a similar 2×3 RM-ANOVA found a main effect of *Type of Effect* on accuracy ( $F_{1,47}=0.12,p=0.02,\eta^{2}=0.001$ ) and an interaction between the factors ( $F_{2,94}=5.89,p<0.001\eta^{2}=0.002$ ). Unlike our previous studies, time-variant effects were more accurate than time-invariant effects. The interaction is largely driven by the superiority of time-variant effects when the display contained three distractor types, with time-variant effects achieving an accuracy of 87% compared to an accuracy of 79% for time-invariant effects.

Second, we looked at the types of time-variance (fixed duration vs variable duration). For search time, a 2×3 RM-ANOVA (*Type of Time-Variance*×*Number of Distractor Types*) showed a significant main effect of *Type of Time-Variance* ( $F_{1,47}=1.78,p=0.001,\eta^{2}=0.0003$ ), but no interaction between the factors ( $F_{2,94}=0.12,p=0.72$ ). Overall, emphasis effects with a fixed duration had faster search time (6467 ms) than variable-duration effects (7235 ms). For accuracy, a similar 2×3 RM-ANOVA found no main effect of *Type of Time-Variance* ( $F_{1,47}=8.75,p=0.17$ ) and no interaction with *Number of Distractor Types* ( $F_{2,94}=0.20,p=0.81$ ).

## Experiment 4: Effects of visualization type on emphasis

Our first three studies all used a basic scatterplot visualization. To explore the robustness of emphasis effects with different kinds of visualizations, our fourth experiment extended our evaluation into four additional visual representations from the Massvis dataset^94^ that have several differences from the basic scatterplot: a dark-mode scatterplot, a Hexbin plot, a Hexbin Map, and an Infographic.

### Stimuli and visualization types

**Figure 14.**
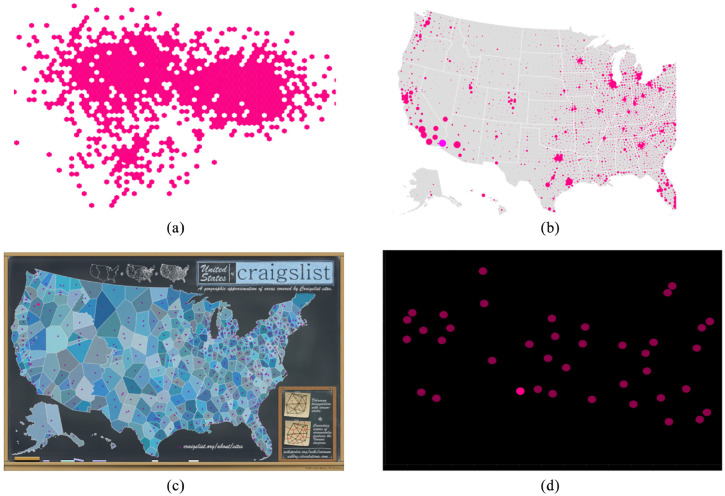
Additional visualization types and backgrounds: (a) Hexbin, with target shown using size in lower left quadrant, (b) HexbinMap, with target shown using color in lower left, (c) Infographic, with target shown using color in the top-left quadrant, and (d) dark-mode scatterplot, with target shown using color in lower middle. We also included the baseline scatterplot from Study 1 (see Figure 1).

We used the same emphasis effects as the other studies, and included the same magnitude levels used for Studies 2 and 3 (1, 3, and 5) giving us coverage of the range we used in the baseline study. We added a new factor, Visualization *Type*, with the five levels described below (and illustrated in Figure 14).

*Baseline scatterplots* are the same representation chosen for Study 1 (distractors were equal-sized filled circles, at low density). An example scatterplot is shown in Figure 1.

*Dark-mode scatterplots* are similar to baseline scatterplots, but use a black background that accentuates contrast.

*Hexbin plots* divide a spatial region into cells (hexagons in our implementation), and color each cell based on a data value. We used a binary data variable, meaning that cells were either filled (variable = 1) or not visible (variable = 0). This meant that all distractors in the visualization were the same shape (hexagon) and the same color. In regions of a Hexbin plot where most cells are filled, large areas of the visualization can be covered; this can mean that emphasis effects using size or motion will overlap other cells (e.g. Figure 14(a)).

*HexbinMaps* are similar to Hexbin plots, but add a map graphic as a background to better indicate the underlying spatial locations of each data point. In our implementation, we also encoded a data variable using size (see Figure 14(b)). For trials that used Size as the emphasis effect, the target size was always calculated from the largest size used for the distractors.

*Infographics* are a stylized representation of spatial regions that can represent multiple variables (e.g. the number and distribution of areas covered by CraigsList sites in the U.S.). The background of an infographic can include arbitrary colors and shapes, and therefore presents a much more complex visual environment than a standard scatterplot.

Our visualization types represent a range of styles that differ in several ways from standard scatterplots. We chose types where all of our emphasis effects could be accurately applied to data in the visualization, without distortion. This meant that some common kinds of visualization could not be used – for example, emphasizing a bar chart with size would distort data perception. Exploration of other visualization types in future work is discussed later in the paper.

### Experimental design

Our experiment used the same binary forced-choice task as in the previous studies, measuring perceived emphasis difference across different magnitude levels and visualization types. We again used accuracy, search time, and visual rating as our primary dependent measures for this study. Each participant completed one trial per *emphasis effect*×*magnitude*×visualization *type* combination (180 total), and 15 trials with no stimulus present (5 trials per visualization type).

### E4 results

We recruited 50 new participants for this experiment, discarding preliminary data from participants who did not complete the study and replacing them with new participants. From the 50 successful completions, we followed the same exclusion criteria described above, resulting in three participants being having an overall completion time outside 3 SD from the mean (one) or for skipping trials and rating questions (two). No participants were excluded from analysis from low accuracy on engagement check trials. The study procedure took approximately 30 min to complete, and participants were compensated with $7 USD for their participation. We analyzed the results from the remaining 47 participants ( $\mu_{\mathrm{age}}$ = 37.47, $\sigma_{\mathrm{age}}$ = 12.47, 25 men, 21 women, 1 non-binary, 2 no response). As above, we report effect sizes as general eta-squared $\eta^{2}$ , and use Holm correction for follow-up tests.

Effects of visualization type on search time: The overall completion time in Study 4 was 7866 ms, which is higher than Study 1 (6872 ms). A 12×3×5 RM-ANOVA (*Emphasis Effect*×*Magnitude of Difference*×Visualization *Type*) showed a significant main effect of Visualization *Type* ( $F_{4,188}=49.55,p<0.001,\eta^{2}=0.02$ ) on search time. Post-hoc t-tests on visualization type pairs found significant differences between all visualization type pairs (all *p* < 0.01) except for HexbinMap and Infographic (*p* = 0.6) and Scatter and Dark mode scatter (*p* = 0.68). The RM-ANOVA also showed main effects of *Emphasis Effect* ( $F_{11,517}=34.41,p<0.001,\eta^{2}=0.04$ ) and *Magnitude of Difference* ( $F_{2,94}=78.45,p<0.001,\eta^{2}=0.01$ ).

**Figure 15.**
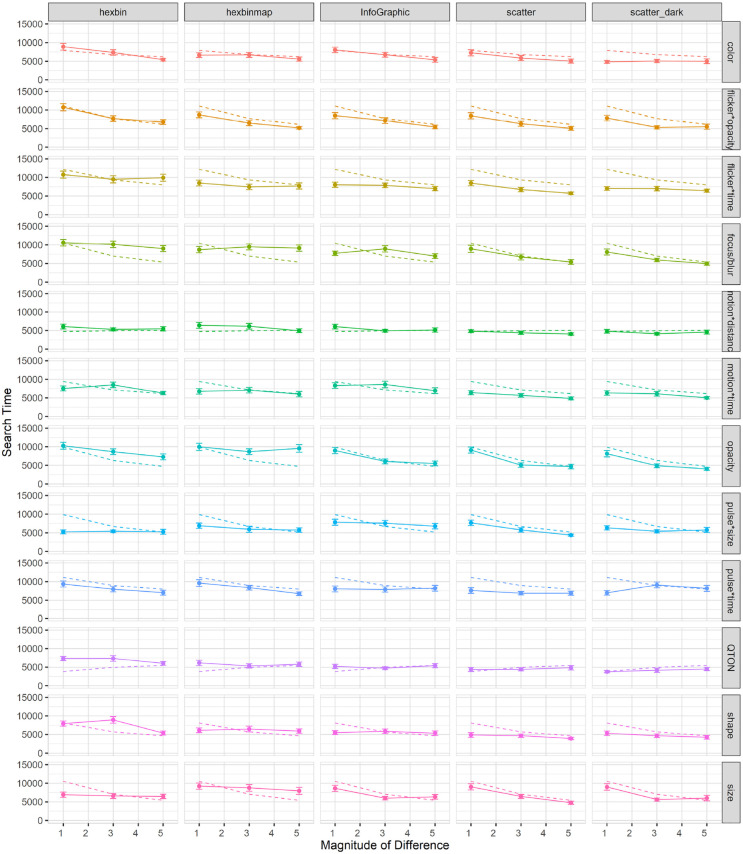
Mean search times (±SE) per variable and visualization type. Dashed lines show predictions from our model.

RM-ANOVA additionally found an interaction between *Emphasis Effect* and Visualization *Type* ( $F_{44,2068}=2.07,p<0.001,\eta^{2}=0.08$ ). As shown in Figure 15, several emphasis effects performed differently in different visualizations. For example, color effects were identified faster with the Dark mode scatterplot than with other types, and Focus/Blur and Flicker effects were slower for Hexbin and HexbinMap. Other emphasis effects were similar across all visualization types (e.g. Motion*distance or Motion*time).

Some results are likely caused by specific differences in the visualization types. First, there was a clear improvement for Color at low magnitudes in the Dark mode scatterplot compared to the baseline scatterplot (search time at magnitude 1 improved from 7252 ms in the white-background scatterplot to 4842 ms in the dark background), a result that is likely due to the improved contrast provided by the dark background. Second, there was a notable difference for Size: in the Hexbin and HexbinMap visualizations, there was almost no improvement with increasing magnitude – unlike the other three visualization types (see Figure 15). This result may relate to Studies 2 and 3, where increased distractor density led to difficulty in estimating size differences, and where increased distractor types interfered with estimates of the same visual variable. Hexbin and HexbinMap have more distractors than the baseline scatterplot (Figure 15), and distractors had multiple sizes in HexbinMap. These factors may have led to the flat response to increasing magnitude, while we see the expected improvement with magnitude in the other visualization types.

Effects of visualization type on accuracy: Overall accuracy in Study 4 was 78%, which implies a false-negative rate of 22%; overall accuracy went down in study 4 compared to study 1 (85%). A 12×3×5 RM-ANOVA showed a significant main effect of Visualization *Type* ( $F_{4,188}=26.09,p<0.001,\eta^{2}=0.01$ ) on accuracy. Post hoc *t*-tests showed that Hexbin had lower accuracy than Hexbinmap, Infographic, Scatter, and Dark mode scatter; and that Infographic had lower accuracy than HexbinMap and Scatterplot (all p<0.001 ). The RM-ANOVA also showed main effects of *Emphasis Effect* ( $F_{11,517}=81.16,p<0.001,\eta^{2}=0.08$ ) and *Magnitude of Difference* ( $F_{2,94}=389.09,p<0.001,\eta^{2}=0.07$ ), similiar to Studies 1–3.

Accuracy on the trials with no target was 83%, implying a false-positive rate of 17%. This rate is similar to Studies 2 and 3, and higher than Study 1 (at 10%).

**Figure 16.**
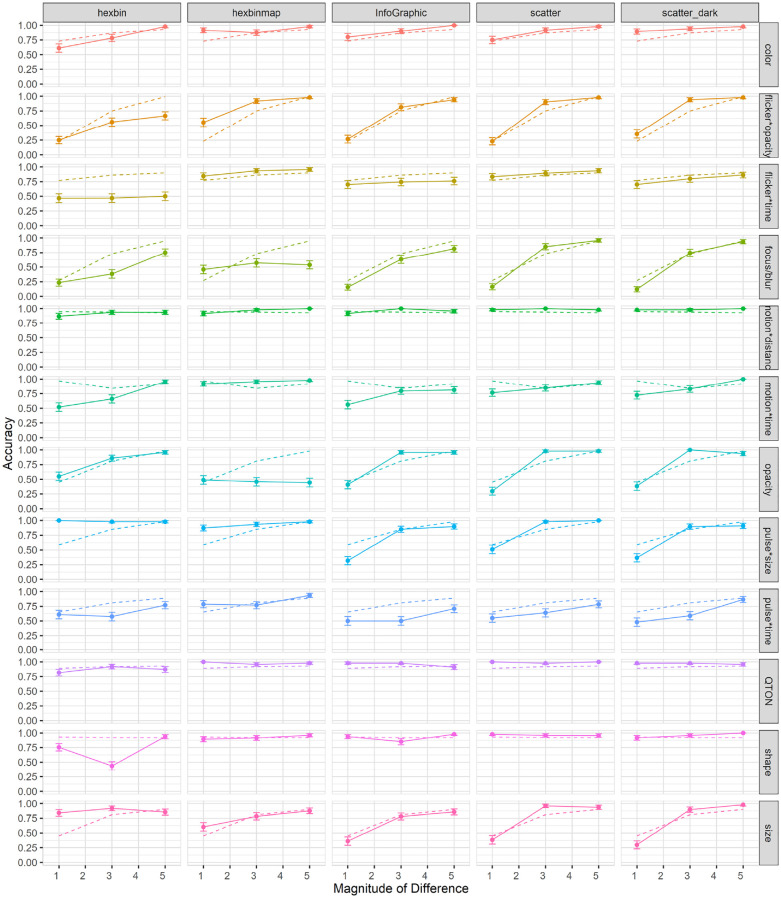
Mean accuracy (±SE) per variable and visualization type. Dashed lines show predictions from our model. .

We also found an interaction between *Emphasis Effect* and Visualization *Type* ( $F_{44,2068}=11.42,p<0.001,\eta^{2}=0.25$ ), between Magnitude and Visualization type ( $F_{8,376}=8.53,p<0.001,\eta^{2}=0.01$ ) and a three-way interaction ( $F_{88,4136}=2.76,p<0.001,\eta^{2}=0.04$ ). These interactions are shown in Figure 16, where several anomalous results can be seen:

- Flicker*Time was substantially less accurate for Hexbin than for the other visualizations;
- Motion*Time was much less accurate than expected at Magnitude 1 for Hexbin and Infographic;
- Pulse*Size was much more accurate at low magnitudes for Hexbin and HexbinMap than for the other visualization types;
- One type of Shape (the filled diamond shape) had particularly low accuracy for Hexbin;
- Opacity was inaccurate at any magnitude for HexbinMap.

Performance for opacity was relatively equal in all visualization types except for HexbinMap, where it had a maximum accuracy of 0.56 across magnitudes. Another notable difference can be seen in Pulse*Size which had a performance ceiling in Hexbin-based visualizations at all magnitudes, while showing an increase with magnitude in the other visualization types.

Contrary to the linear performance seen in Figure 15 for Size’s search times, we see an increase of performance in accuracy across magnitudes in the Hexbin-based visualizations. This result suggests that while search time remained relatively unchanged across magnitudes, size judgments became more accurate as magnitude increased.

**Figure 17.**
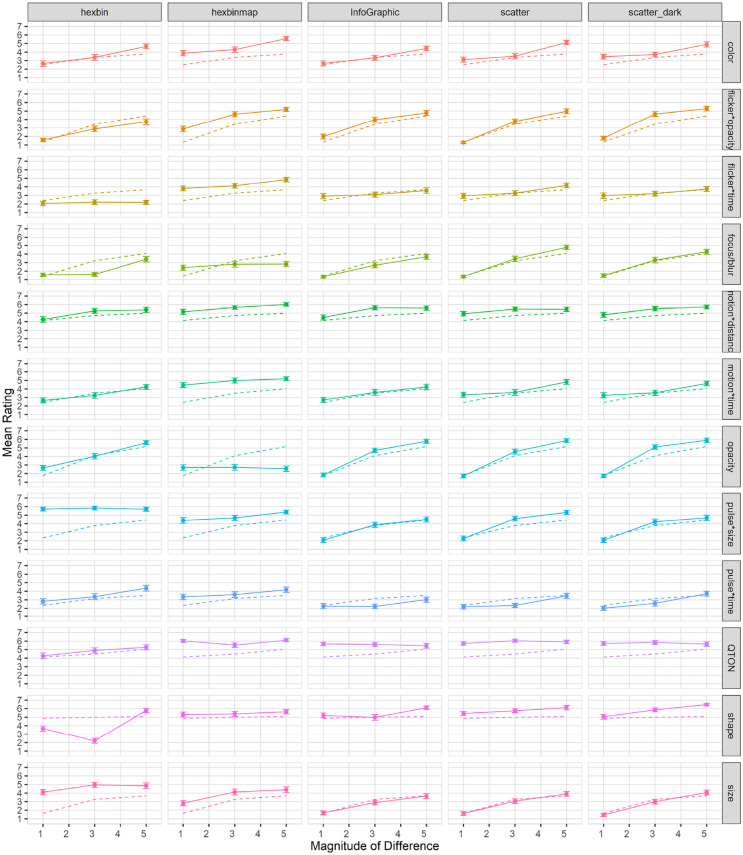
Mean rating (±SE) per variable and visualization type. Dashed lines show predictions from our model.

Effects of visualization type on subjective perception of magnitude of difference: As shown in Figure 17, mean rating scores followed a trend similar to accuracy. RM-ANOVA using the Aligned Rank Transform showed significant effects of Visualization Type, Emphasis Effect, and Magnitude on subjective ratings (all p<0.001 ) as well as an interaction between *Emphasis Effect* and Visualization *Type*. The rating results essentially mirrored those of accuracy, suggesting a strong correlation between accuracy results and subjective responses.

### Exploratory analyses

Similar to the previous three studies, we carried out a series of analyses as a follow-ups, looking into how visualization type affected the different groups of emphasis effects. A 2×5 RM-ANOVA found no significant main effect of *Type of Effect* ( $F_{1,46}=1.84,p=0.17$ ) on search time, but found an interaction between *Type of Effect* and Visualization *Type* ( $F_{4,184}=3.85,p<0.001,\eta^{2}=0.003$ ). This interaction is largely guided by time-variant effects being faster in visualizations that have more complex visual elements (Hexbin, HexbinMap, and Infographic): the average search time for time-variant effects across these three types was 7211 ms, compared to time-invariant effects requiring a search time of 7309 ms. Conversely, time-invariant effects were found to be faster in both types of scatterplot (search time of 5665 ms, compared to time-variant effects at 6178 ms). For accuracy, a 2×5 RM-ANOVA found no main effect of *Type of Effect* on accuracy ( $F_{1,46}=0.115,p=0.7$ ), but did find an interaction between *Type of Effect* and Visualization *Type* ( $F_{4,184}=17.53,p<0.001,\eta^{2}=0.008$ ).

Second, we again considered the types of time variance, and divided the variable into two groups (constant-duration or variable-duration). A 1×2 RM-ANOVA found a significant main effect of *Type of Time-Variance* ( $F_{1,46}=98.68,p=0.001,\eta^{2}=0.02$ ) on search time but no interaction between the factors ( $F_{4,184}=1.70,p=0.14$ ). Overall, variables with a constant duration had a faster search time of 6095 ms, compared to variable-duration effects requiring an average of 7501 ms. A 2×5 RM-ANOVA found a significant main effect of *Type of Time-Variance* on accuracy ( $F_{1,46}=40.23,p=0.001,\eta^{2}=0.003\eta^{2}=0.009$ ), with constant-duration variables (e.g. pulse*size, motion*pixels, and flicker*opacity) having a higher accuracy (82%) compared to variable-duration (74%). The RM-ANOVA also found an interaction between *Type of Time-Variance* and Visualization *Type* ( $F_{4,184}=6.00,p<0.001,\eta^{2}=0.005$ ), with constant-duration variables outperforming variable-duration for all visualization types.

### Model consistency across visualization types

We used the models built from Study 1 data to predict the data gathered for each visualization type, and then compared the empirical data points to the predicted values (predictions are shown in Figures 4–6 as dotted lines). Many predictions had correlations as high as 0.99 (e.g. color in infographic, or flicker*time in Hexbin). Although in most cases there was a high correlation between the predictions and the actual values, we found some cases where emphasis effects differed from their prediction. One such case was color when presented in a dark-mode scatterplot, where our prediction had a correlation of 0.039. Although the value of the predictions differed, the predictions do capture many of the characteristics and differences among visualization types and offer a way to evaluate further visualization studies. Ultimately, we tested the correlation between the predicted and empirical values for all variables except for shape: for search time, the correlation was 0.68 (SD 0.33); for accuracy, correlation was 0.67 (SD 0.33); for subjective ratings, we had the highest correlation of 0.84 (SD 0.20).

## Discussion

The four studies reported above investigated how users perceive 12 emphasis effects in a variety of viewing conditions – including several magnitude levels, scenarios with a variety of distractors and distractor types, and a range of more complex visualizations. In the following sections, we first return to our initial question of “which emphasis technique to use” and summarize our results for each emphasis effect. See Table 2 for an overview. We then identify generalizable findings from the four studies, provide explanations for these general findings, and discuss ways that designers can make use of our results.

**Table 2:** Summary of key takeways per variable in each study starting from baseline magnitude (Study 1).

	Baseline magnitude	Distractor amounts	Distractor types	Visualization types
Color	High Acc starting at low Mag.	No differences.	No differences.	No effect of Mags in HexbinMap
Size/area	50% Acc at mag 1. High Acc Mags 2+	Slower search time at Mag 5	No differences.	High Acc in Hexbin.
Blur/focus	Worst Mag 1. Ceiling at Mag. 4	Slower search time at Mag 1	Increased Acc in lv3 at Mag 1	No differences.
Opacity	Low Acc at Mag 1. Reaches ceiling at Mag 3.	No differences.	No differences.	Low Acc all Mags in HexbinMap
QTONs	High performance, all Mags	No differences.	No differences.	No differences.
Shape	High performance, all shapes	No differences.	Slower search time and lower Acc	Low Acc filled diamond in Hexbin
Motion × time	73% Acc at Mag 1. Reaches ceiling at Mag 5.	No differences.	Increased Acc in lv3 at Mag 1	Low Acc Mag 1 Hexbin and Infographic
Motion × distance	Performance ceiling at all Mags	No differences.	No differences.	No differences.
Flicker × time	75% or higher Acc at all Mags	No differences.	No differences.	Low Acc in Hexbin
Flicker × opacity	Low Acc at Mags 1,2. 80%+ at Mag 3–6	No differences.	Increased Acc in lv3 at Mag 1	No differences.
Pulse × time	Starting 65% Acc at Mag 1	No differences.	Increased Acc with 3 distractor types	No differences.
Pulse × size	High Acc at Mags 2+	Slower search times	Increased Acc with 3 distractor types	Most Acc at low Mags in Hexbins

Acc: accuracy; Mag(s): magnitude(s).

Comparisons across studies are in relation to the baseline results.

### Summary of results by emphasis effect

#### Color

In Studies 1 and 2, Color was similar to other static effects in terms of search time (other than Shape, as discussed below), but showed higher accuracy at low magnitudes than focus/blur, opacity, or size (accuracy with Color was at or above 75% even at magnitude 1 in both studies). Study 3 showed that perception of Color was not affected by increasing the number of distractor shapes. In Study 4, Color performed similarly to the earlier studies, with comparable search times to other static effects and high accuracy even at low magnitudes. The additional background colors in the Infographic visualization did not negatively affect either search time or accuracy. In addition, Color performed particularly well in the dark-mode scatterplot, with lower search times and higher accuracies than the white-background scatterplot.

Overall, these results show that Color is an effective and consistent emphasis effect across a variety of viewing conditions – even with different background colors, and particularly in settings where contrast with the background is high. We note, however, that we screened participants for color-vision deficiencies, and in real-world use designers must ensure that emphasis colors are differentiable. In addition, when designers use color to encode additional variables, the ability of color to provide pop-out emphasis is strongly constrained.^3^

#### Shape

Study 1 showed that Shape was perceived quickly and accurately, regardless of the target shape used. However, Shape was different from the other effect types in that we tested five different shapes rather than a true series of magnitude levels (e.g. a gradual morph from the distractor shape to the target shape as was done in earlier studies^4^). Our intention was to determine whether different shapes have differences in terms of their perceptibility – but this meant that each of our tested shapes was already at its maximum difference from the distractor shape. We did not see any clear ordering of the different shapes in terms of perceptibility, and all shapes performed well in Study 1. In Study 2, all shapes were again perceived equally quickly; however, our accuracy results show that the filled diamond and the unfilled star may have been more affected by clutter than the filled square.

Study 3 was of particular interest for Shape because this study varied the number of shapes in the distractors. Although Shape performed comparably to the other effects, search times with the filled diamond shape were more affected by the increasing number of distractor types, and accuracy was more affected for the filled square and the filled diamond.

**Figure 18.**
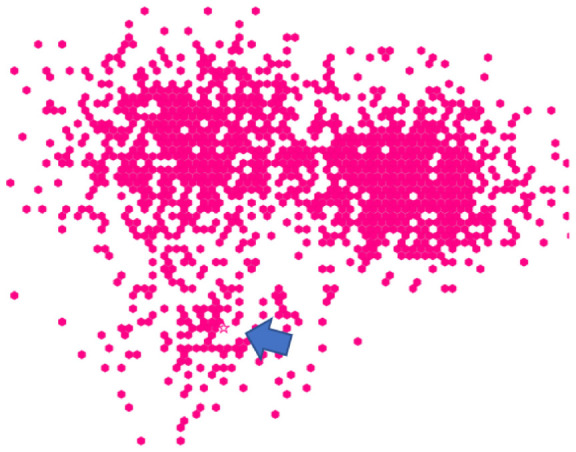
Overlaping and crowding among distractor items in Hexbin increase difficulty for emphasis effects. Shape (unfilled star) is present near the arrow (added through photo-editing software).

In Study 4, there was an obvious anomaly in the overall strong performance of Shape – that is, the poor accuracy results of Shape in the Hexbin visualization. This result appears to be due to the overlap that is caused when manipulating shape within the space-filling layout of the Hexbin; that is, the different shapes were difficult to see because of overlap between data items (see Figure 18).

Although our results show that shape is a poor choice for space-filling layouts (and likely for any crowded layout where the characteristic visual elements of different shapes will be more difficult to discern), shape was a strong emphasis effect – it was one of the top performers in almost every setting, and was even comparable to other effects when there were multiple distractor shapes. We recommend shape for emphasis in any visualization other than those that use a space-filling layout; shape can also be an alternative to motion when animation is not possible.

#### QTON

Across all studies, results showed that QTONs can be perceived quickly and accurately, regardless of magnitude level. In Study 3, however, we saw a decrease in performance when multiple distractor types were present, and similar to Shape, in Study 4, we saw a minor decrease in performance for QTONs in the Hexbin’s space-filling layout.

QTONs are a set of shapes that were designed to be perceptually ordered – that is, shape in the sequence is visually distinct and is perceived as increasing or decreasing in value. Our QTONs start with an unfilled circle with one intersecting line, moving to a circle that is almost filled by lines at level 5, giving us a progression from unfilled to filled (distractors were unfilled circles). While QTONs were designed to be perceptually ordered, we must note that the changes are often minimal, such that there is little perceptual difference between each QTON in the sequence. While we expected that QTONs would be progressively harder to identify as they got closer in similarity to the filled circle, our results show performance that replicates that of shape at any magnitude level. That is, viewers identified each shape in the sequence of QTONs as individual shapes, rather than as an effect that progressively changed based on magnitude level.

Overall, QTONs were a strong emphasis effect across all settings, with our results showing a decrease in performance only for dense layouts (e.g. Hexbin), or when distractors used multiple shapes. Similar to Shape, QTONS are an alternative to equally perceptible motion effects when animation is not possible.

#### Size

In Study 1, Size was similar to other static effects in terms of search time, with a gradual increase in performance at each increase of magnitude level. In study 2, perception of Size (and its time-varying counterpart Pulse*Size) appeared to be more affected by clutter than other effects. While we ensured that distractor positions did not cause overlaps, it is possible that an overall higher numbers of objects made differences in size more difficult to perceive. Results for Size in study 3 largely mirror those of study 1: however, for pulse*size, accuracy actually increased (from 0.48 to 0.82) when more distractor types were present. Finally, in study 4, Size showed almost no improvement with increasing magnitude for the Hexbin and HexbinMap visualizations.

Overall, Size was a strong emphasis effect at a higher magnitude level in simple visualization types, but had limited effectiveness in visualizations that were crowded or dense. In visualizations where size was clearly visible, we found that its performance was strongly predicted by magnitude level, which offers designers a way to precisely control perceived importance of a data point. For maximum effectiveness, designers must use a high magnitude level, and must check whether the increased size leads to overlaps with other items in the visualization – if so, it is recommended to use a different emphasis effect.

#### Opacity

Opacity (along with Blur/Focus) was different from other effects in that it applies its visual change to all of the elements of the visualization *except* the emphasized (target) point. Even with this substantial overall difference, however, study 1 showed that Opacity largely followed the results of the static effect Size, and that the performance of Opacity was largely predicted by magnitude with strong emphasis at higher magnitude levels. These results were maintained in studies 2 and 3: the global effect of Opacity leaves the target as the most visible object on the screen, explaining the effect’s resilience to an increasing number of distractors or multiple distractor types. This performance largely carried over to study 4, where Opacity performed well for all visualization types except Hexbinmap. This anomaly can be partially explained by the gray background map in the HexbinMap visualization: because our opacity filter affected only the distractor items and not the map itself, it is possible that participants were distracted by visual features of the background (since these were not affected by the opacity filter).

Our results show that opacity is a consistent emphasis effect across a variety of viewing conditions, but designers must ensure that any object in the background that can be an interference between target and distractors is also properly affected by the opacity filter to avoid the results we saw in the HexbinMap visualization. As an effect that essentially hides everything except the object in focus, opacity can be used in most situations. However, it may not be suitable when the user needs to compare items, since these may be affected by the filter.

#### Blur/Focus

Blur/Focus is another technique that affects all elements of a visualization except the target; and similar to Opacity, results from study 1 showed that Blur/Focus performance is strongly predicted by magnitude level. Blur/Focus was the worst performer of all the emphasis effects at magnitude 1, but was above 90% accuracy at magnitude 4 and above. These results were maintained across study 2, but in study 3 our results showed poor accuracy of Blur/Focus at low magnitudes (e.g. 0.14 at magnitude 1 with one distractor type and 0.52 with three distractor types). The results for study 4 across visualization types followed the same trend as those for Opacity, in which an increase in magnitude had no effect on perceptibility for Blur/Focus in HexbinMap. Similar to opacity, the blur effect was applied only to the distractor data points and not the background map, so increasing magnitude still left the background map as an object that could visually interfere with the target.

Our results indicate that Blur/Focus can be a strong alternative to other emphasis effects, however, it must be used at a higher magnitude level to achieve a comparable performance. Care must be taken so that any object which may interfere with a target has the blur filter properly applied, as it may diminish a viewer’s ability to perceive the target. Similar to Opacity, Blur/Focus can be used in a variety of scenarios, and it can be interchanged with opacity if the opacity filter is not available.

#### Time-variant effects

The results from study 1 and 2 show that identifying time-variant effects was slower than time-invariant (static) effects, but we found no differences for accuracy. Within types of time-variant effects, we found that effects with constant duration (i.e. that varied the magnitude of the visual variable) were faster than variable-duration effects (that held constant the magnitude of the visual variable). We found no differences between types of time-variant effects in study 1, but in study 2 we found that constant-time effects were more accurate. We attribute these findings to the fact that time-invariant effects contained Shape and QTONs, which both showed strong performance at all levels, while most time-variant effects had a gradual increase in performance as magnitude increased.

In study 3, and unlike our previous studies, time-variant effects were more accurate than time-invariant effects. This result was guided by the superiority of time-variant effects when the display contained three distractor types, with time-variant effects achieving an accuracy of 87% compared to an accuracy of 79% for time-invariant effects. We again found fixed-duration effects to have a faster performance. In Study 4, time-variant effects were found to be faster in more complex visualizations (such as Hexbin and HexbinMap), while static effects were faster in the scatterplots. Similar to our previous studies, we found constant-duration effects to be faster and more accurate than variable-duration effects.

These results show that in most simple scenarios such as basic scatterplots, time-invariant effects will offer better performance. However, the strength of time-variant effects can be seen in more complex visualizations such as scatterplots containing multiple distractor types, or in dense visualization layouts such as Hexbin and Hexbinmap. We recommend using time-variant effects in situations where it can be difficult to identify a single object due to density of distractors in a visualization.

### General findings across the four studies

Our studies showed several findings that were common across some or all of the experiments:

- *Magnitude affects perceptibility.* We found that accuracy improved and trial times decreased as magnitude increased for most variables. This result agrees with those of previous evaluations^48^ and we extend the evaluation to a larger variety of emphasis effects and viewing scenarios. Varying magnitude provides a gradual way to control emphasis perception. Variables that did not exhibit a performance boost by an increased magnitude were at a performance ceiling even at low levels (e.g. QTONs).
- *Differences exist across effects and viewing scenarios*. We saw differences among various effects in all studies, and these differences were often maintained across different scenarios. Designers can use our results to choose an appropriate effect according to specific viewing conditions.
- *Commonalities exist across emphasis effects.* All studies found commonalities across several visual variables, particularly for similar types of effects (e.g. time-variant effects were similar to one another). We found that certain time-invariant effects such as focus/blur can offer a similar experience to animated effects when animation is not possible.
- *Increased distractor types decreased perceptibility for certain effects.* Having different types of distractors made certain effects harder to notice, particularly at lower magnitude levels. Designers should consider using a higher magnitude or use a different emphasis effect to ensure perceptiblity in scenarios with multiple distractor types.
- Visualization *clutter affects size perception.* Increasing the number of distractors significantly affected trial times for size-based emphasis effects. However, accuracy and visual rating remained unaffected by clutter amount.
- *Commonalities exist across* visualization *types.* We tested our emphasis effects in a range of visualization types and backgrounds. While this analysis needs to be extended to more visualization types, we found similarities among several visualizations, suggesting that perceptibility results from simple scatterplots can be extended to other visualization types. These commonalities are further exemplified by a high correlation between predicted responses from our model and actual values from the study.

In the following sections, we consider explanations for these main results, explore how our findings can assist visualization designers, and discuss directions for future research.

### Explanation of results

#### Contrasting performance with increased distractor types

Work by Haroz and Whitney^31^ had previously found that in a task with a random ordering of an “oddball” target, performance degraded as variety of visual elements increased. Haroz and Whitney’s work largely focused on color and motion as their primary effects. We found that the added distractor types made certain effects (particularly pulse*size and focus/blur) easier to notice particularly at magnitude level 1 (e.g. pulse*size had an accuracy of 0.48 with one distractor type compared to 0.82 with all three distractor types, and focus/blur had an accuracy of 0.14 at magnitude 1 with one distractor type compared to 0.52 with three distractor types). Search times, however, were largely unaffected by added distractor types for most emphasis effects, with a minor increase in search time for some variables such as opacity and motion*distance.

The effects that performed well may have been helped by viewer’s ability to group similar objects. According to visual grouping theory, objects that are similar in nature (such as size, shape, or color) tend to be grouped together.^18,95^ In our experiment, viewers may have formed groups of distractors based on shape, and additionally based on size. If a visualization uses size or focus as emphasis, the target may be more easily discernible as different from any group of stimuli, thereby increasing accuracy for the emphasized target.

#### Differences in size perceptibility across tasks

While previous work has evaluated a limited range of visual features^4,20^ and has found them to be effective at guiding attention in scatterplot visualizations,^47,48^ it is known that insufficient distinctiveness and background complexity can degrade results.^13,92,96^

Perceptibility results in our task with an increased number of distractors (rather than distractor types) show that increasing distractors primarily affects those emphasis effects that varied size (e.g. Pulse). Recent work suggests that size is not completely separable from other variables such as color.^29,58,67^ An increased number of distractors, leading to more elements and a larger portion of the visualization space filled by the color in the distractor elements could have lead to the increased difficulty in detecting size differences.

As described above, effects that varied the size of an element were less prominent than expected, which we attribute to visual crowding from other elements in the visualizations, and reduced separability of size from other visual variables. While size perceptibility was generally robust in our baseline study, size performance degraded as more complexity was added to visualizations (e.g. number of distractor types or different visualization styles). This result indicates that there can be emergent properties in certain real-world visualizations scenarios that interfere with the user’s perception of emphasis, and thus a planned emphasis effect must be considered in light of other visual elements and their interactions.

#### Effectiveness of the predictive model

The model built from Study 1 data provided reasonably accurate predictions for many results across visualization types in Study 4 ( $R^{2}$ values of 0.67 (SD 0.33), 0.68 (SD 0.33), and 0.83 (SD 0.20) for trial times, accuracy, and subjective ratings). The predictive model shows that perception of emphasis is at least somewhat consistent across various visualization types. In addition, the model was a useful tool for identifying individual differences of an effect across visualization types (e.g. color in different backgrounds). We also note that the model had the highest correlation in predicting subjective visual ratings. Ultimately, designers want to know how emphasis will be experienced by viewers, and visual ratings offer the best insights about how emphasis is perceived.

#### Experimental factors

We did not see a significant decrease in performance of Shape with increasing distractor types. However, this could have been influenced by our choice of tested shapes, as we saw a drop in accuracy for the filled diamond shape from 93% with one distractor to 69% with two background distractors. Future studies should explore the separability between a wider variety of shapes in regards to background distractor types.

There is also the possibility of noise within our results, particularly for reaction times. Browser configurations, anti-aliasing, display differences, and hardware settings could have affected the way visualizations were rendered, while bandwidth differences could have affected how quickly they were delivered to our participants, potentially affecting reaction times. Crowdsourcing represents a trade-off of control and ecological validity – it allows us to model visualization perception in real-world contexts and has been shown to be effective in previous work, although it increases the number of uncontrolled random variables.^24,29,35,41,63,64^

### Implications for designers and generalizing the results

Our goal is to improve visualization effectiveness in the real world; our studies improve understanding of how visual cues are detected as emphasis effects, offering insights into their perceived visual prominence. Crowdsourcing our studies allowed us to evaluate emphasis effects in a wide variety of display and browser settings, and allowed us to extend our findings to a range of different visualization contexts. While our results are directly applicable to visualization designers who need to draw a user’s attention to important data points, it is important to consider how these results might also affect higher-level tasks, such as identifying correlations or averages.

The measures we used to evaluate visual stimuli offer insights as to how emphasis effects are experienced by viewers. Search times relate to how fast a viewer can process a potentially salient element in a visualization while accuracy offers insights into viewer’s conscious decision as to whether an element appears to be emphasized, and visual ratings offer confirmation of this conscious decision by evaluating the chosen emphasized element. Across all studies, we found that accuracy and subjective ratings were closely aligned (although for ratings, the scale was compressed). When designers emphasize an element, they typically want the viewer to know that the item is being emphasized. As accuracy and visual rating relate to a viewer’s conscious decision about an element, these two measures are possibly a better way to analyze and model experimental data: ultimately, what the viewer thinks is being emphasized is possibly more important than what their attention is guided to first.

*Emphasis cues based on chart and display configurations*: A first design implication is that time-variant (animated) effects such as motion*time can achieve a high perceived visual prominence and remain relatively unaffected by a visualization’s background. These results are in accordance with previous work by Bartram and Ware^8,28^; however, rather than suggesting motion for all situations, we encourage designers to consider other factors. Animations in visualizations are not always possible in media such as print or when limitations exist due to browser settings and hardware configurations. In addition, motion-based effects are not appropriate when the user needs to see the actual position of the emphasized element.

Our results show that some time-invariant effects can achieve similarly high perceptibility when presented at a higher magnitude level (such as focus/blur at magnitudes 4 and above). Previous work suggest that size is a separable channel^93^ such that differences in size should be easy to detect. Our results, however, suggest that certain display configurations (such as a crowded background) can affect size perception. We found that shapes and QTONS can also exhibit high performance that is comparable to that of time-based effects, making them good candidates for crowded displays. Designers can utilize our results to ensure a similar experience with different effects across media types and display settings. In addition to these general recommendations, the following effect-specific design implications should be taken into consideration:

- Size differences may be difficult to estimate in cluttered/dense visualizations. We saw a drop in search time for size-based effects in a cluttered scatterplot, and no improvement in search time with an increase in magnitude in study 4 with Hexbin and Hexbinmap (which have denser layouts). In these settings, designers can use color to differentiate a specific data point, since color was unaffected by clutter in our study when used at a medium-high magnitude.
- Shape is hard to identify when the visualization involves multiple shape types. Alternatively, pulse*size was easier to notice with more distractor types. This result may be explained by the grouping theory, where the distractor types can be grouped based on similarity – thus an animated change in size would be relatively easier to perceive as different.^11^
- Time-variant (animated) effects based on a constant duration (where the endpoint of the visual variable is varied, rather than changing the duration of the effect), were found to be easier to detect across our studies, making them a more appropriate choice when animated visual effects are used.
- Color performance was improved in the scatterplot with a dark mode background, as it offered a greater contrast that allowed differences in color to be amplified. This results makes color a better choice when users elect to view visualizations in dark mode. Alternatively, color can be preferable to other effects such as motion or size when screen real-estate is limited (such as in mobile phones or smart watches^97^).
- Accuracy for motion-based effects was found to be the most resilient among any kind of background or distractor type. We often found color at a high magnitude to be similarly strong; designers can safely interchange the use of color and motion effects to ensure a similar experience when emphasizing depending on the setting (print vs online) and the characteristics of the user (e.g. with or without a color vision deficiency).

*Magnitude for value comparisons in* visualization: Our results show that certain visual variables such as Focus/blur and Size have a gradual increase in perceptibility, while flicker*time maintains a consistently high perceptibility. The difference between the magnitude levels of these effects can be used in value comparisons following Hall’s et al.^10^ emphasis framework, where variables in a visualization can be assigned to a background B , midground M , foreground F set utilizing emphasis effects to signify importance.

A designer can utilize blur to assign non-important points to the background set, points with increased performance to an un-blurred midground set M , and flicker*time (one of the strongest-performing emphasis effects) can be assigned to assign the value of highest importance or interest in the foreground F set. While performance differences exist at lower magnitude levels, most variables (even classes of variables, e.g. static vs non-static), will have similar performance at a given magnitude level (for ex, level 5 pulse and level 2 motion*distance) – allowing designers to compare and choose a desired effect’s perciptibility based on a magnitude level.

*Emphasis beyond* visualization: Beyond visualization, these results can be extended to user interface design. For example, it is known that humans are not well adapted for tasks that require continuous monitoring^93^; therefore, designers may choose different levels of visual perceptibility to represent different types of notifications (e.g. a highly perceptible motion alert for an emergency notification, vs a low-magnitude color difference for an email notification), emphasizing icons in a desktop environment based on importance or usage, or text highlighting in document readers.

Designers want to ensure emphasis effects are experienced in a similar way across different contexts. This is especially important for viewers with color-vision deficiencies (CVDs) – and we note that some CVDs can affect ordinary users because they arise from changes in the environment (e.g. looking at a mobile device in bright sunlight, or through colored glasses, strongly alters color differentiability^98^). To account for CVDs, designers may use a similar method to ours to measure perception of color difference in CVD-accessible color palettes. Furthermore, designers may use our metrics and expand on our results by evaluating the effectiveness of different visual effects with users who have color-vision deficiencies to ensure the same perceived importance.^47,99^ Similarly, our current evaluation does not take into account any potential learning or developmental differences in our participants,^99^ however, future studies can utilize our methods and extend our models so visualization designers have more options for creating accessible visualizations (see Wu et al.^99^ for a review on designing accessible visualizations).

### Limitations and future work

The difference levels for the visual variables tested in our experiments are intended to be generalizable for the design of emphasized elements in typical visualizations. As there is currently no equivalence metric between the 12 effects we tested, our findings are influenced by the magnitude of differences we tested and the range of difference that is possible with each visual variable. We additionally varied distractor number and types, but within this context, we tested only a single emphasized data point. An opportunity to extend to our work is to investigate visualizations that emphasize multiple points at once – an important step for deriving the usefulness of emphasis for tasks such as value comparisons – and extend this evaluation to include more distractor variations (e.g. varying shape and size along with color and opacity) and test the limits of emphasis by testing combinations of distractor amounts, types, and other emphasis effects. Furthermore, these combinations can then be evaluated within the context of multiple visualization types and backgrounds.

Our third study varied the number of distractor shapes. While we found most variables were not affected by including up to three distractor types, future studies can extend this evaluation to include more distractor variations (e.g. varying shape and size along with color and opacity) and test the limits of emphasis by testing combinations of distractor amounts, types, and other emphasis effects. We elected to use CIELAB for our color difference metrics; however, future work can expand to other color difference models or color spaces, such as CIECAM02.^100^ Additionally, while we found no effect of distractor type for Shape, these results may be influenced by the specific shapes we used for our evaluation. We found that accuracy for the filled diamond shape decreased, and it is possible that other shapes will be similarly affected by having multiple background distractor types. Future studies can categorize shape types (e.g. open and closed shapes) or shapes that closely align to known icons,^101^ evaluate interactions with emergent and asymmetric popout patterns, and evaluate which type of shapes are most affected by background distractors.

Finally, we did not consider the distraction that could be caused by over-emphasizing an element. Previous work suggests that there can be an interference between the visual features of a primary task and those used to draw attention to elements.^20^ While most previous research focuses on interruption caused by alerts and notifications, attending to an emphasized element may interrupt an analysis task when viewing visualizations, which can have a detrimental effect on user performance.^102^ Because over-emphasizing elements may distract viewers from their analysis tasks, future work should explore the interplay between emphasis and distraction.

## Conclusion

Emphasis is an essential component of information visualization, and is used by designers to draw a user’s attention or to indicate importance. However, it is difficult for designers to ensure a similar experience with different emphasis effects in a wide range of contexts and viewing scenarios. Through a series of crowdsourced studies, we evaluated perceived prominence of twelve emphasis effects in scenarios that varied distractor amounts, distractor types, and visualization types and backgrounds. Our studies showed that while commonalities exist among various effects, perceived prominence varies in different contexts. Results from our studies and our predictive model improve our understanding of how visual effects operate and how they are experienced by viewers. These findings provide useful information for designers who want to ensure a similar experience with emphasis effects in a wider variety of contexts, and they contribute to a growing vocabulary for the application of visual stimuli for emphasis in visualization design.

## Supplemental Material

sj-pdf-1-ivi-10.1177_14738716211045354 – Supplemental material for Which emphasis technique to use? Perception of emphasis techniques with varying distractors, backgrounds, and visualization types Click here for additional data file. Supplemental material, sj-pdf-1-ivi-10.1177_14738716211045354 for Which emphasis technique to use? Perception of emphasis techniques with varying distractors, backgrounds, and visualization types by Aristides Mairena, Carl Gutwin and Andy Cockburn in Information Visualization
