# Supplementary material for: Which emphasis technique to use? Perception of emphasis techniques with varying distractors, backgrounds, and visualization types
Source: Inf Vis. 2021 Sep 22;21(2):95–129. doi: 10.1177/14738716211045354 (PMC8841630; doi:10.1177/14738716211045354)
Supplement: sj-pdf-1-ivi-10.1177_14738716211045354 – Supplemental material for Which emphasis technique to use? Perception of emphasis techniques with varying distractors, backgrounds, and visualization types [file sj-pdf-1-ivi-10.1177_14738716211045354.pdf]

Supplemental Material for: Which Emphasis Technique to Use? Perception of Emphasis Techniques with Varying Distractors, Backgrounds, and Visualization Types

Journal Title
XX(X):1–3

©The Author(s) 2021
Reprints and permission:
sagepub.co.uk/journalsPermissions.nav

DOI: 10.1177/ToBeAssigned
www.sagepub.com/
SAGE

Removed for Review[1]

Abstract

The main text provides the results from completed submissions to the studies with participants removed according to a pre-registered methodology. In this appendix, we are providing an analysis of our data that includes removed participants. The inclusion of the removed participants (five in study 1, two in studies 2 and 3, and three in study 4) did not change our main results and conclusions.

Experiment 1: Establishing a Baseline of Perceived Emphasis

Each participant completed 184 trials: two trials per emphasis effect x magnitude combination (144 total), and 40 trials with no stimulus present (as a manipulation check). The main text presents the analysis from 45 successful study completions. The analysis below includes 5 removed participants for reasons including failing to correctly answer most of the engagement checks including skipping trials or rating questions (four) or having an overall completion time outside 3 s.d (one) from the mean.

Results

Search Time: A 12 x 6 RM-ANOVA showed significant main effects of both *Emphasis Effect* ($F_{11,539} = 18.36, p < 0.001, \eta^2 = 0.02$), and *Magnitude of Difference* ($F_{5,245} = 163.95, p < 0.001, \eta^2 = 0.10$) on search time, and an interaction between the factors ($F_{55,2695} = 12.35, p < 0.001, \eta^2 = 0.07$). The data are summarized in Fig 1.

Accuracy: A 12 x 6 RM-ANOVA showed significant main effects of both *Emphasis Effect* ($F_{11,539} = 16.30, p < 0.001, \eta^2 = 0.02$), and *Magnitude of Difference* ($F_{5,245} = 32.95, p < 0.001, \eta^2 = 0.0210$) on search time, and an interaction between the factors ($F_{55,2695} = 2.28, p < 0.001, \eta^2 = 0.01$). The results are summarized in Fig 2.

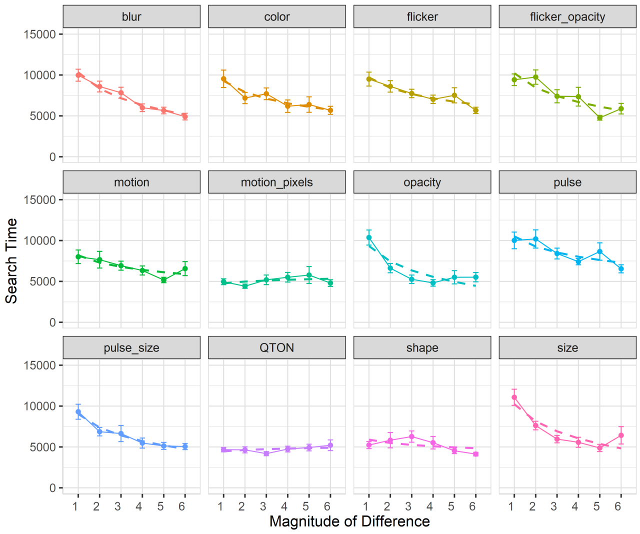

Figure 1. Experiment 1, Mean search times (±s.e.) per Variable. Level one (solid lines), levels 2-3 (dashed lines)

Experiment 2: Effect of Number of Distractors on Emphasis

Each participant completed 153 trials: one trial per combination of *emphasis effect* x *magnitude* x *clutterLevel* (108 total), and 45 trials with no stimulus present (15 trials per clutter level). The main text presents the analysis from 48 successful study completions. The analysis below includes 2 removed participants for reasons including failing to correctly answer most of the engagement checks including skipping some trials and rating questions.

Results

Search Time: A 12 x 3 x 3 RM-ANOVA (*Emphasis Effect* x *Magnitude of Difference* x *ClutterLevel*) showed a significant main effect of Clutter Level on search time ($F_{2,98} = 14.98, p < 0.001, \eta^2 = 0.006$), and also showed effects of Emphasis Effect ($F_{10,490} = 6.93, p < 0.001, \eta^2 = 0.01$) and Magnitude of Difference ($F_{2,98} = 29.01, p < 0.001, \eta^2 = 0.01$). The results are summarized in Fig 3.

[1]Removed for Review
[2]Removed for review

Corresponding author:
Removed for Review
Email: Removed for Review

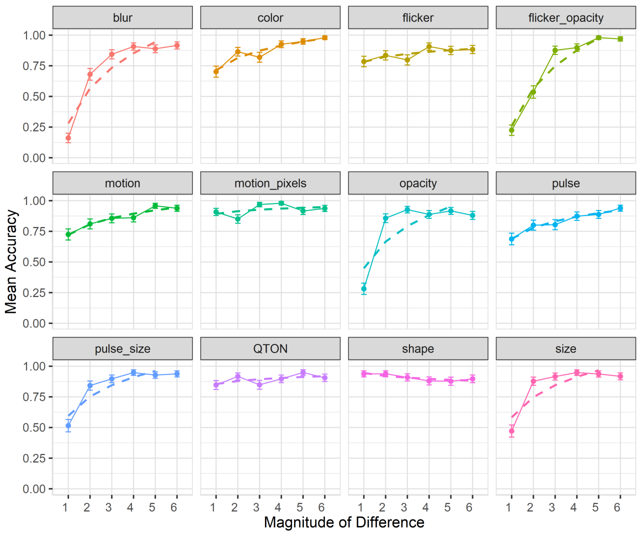

Figure 2. Experiment 1, Mean Accuracy (±s.e.) per Variable. Level one (solid lines), levels 2-3 (dashed lines)

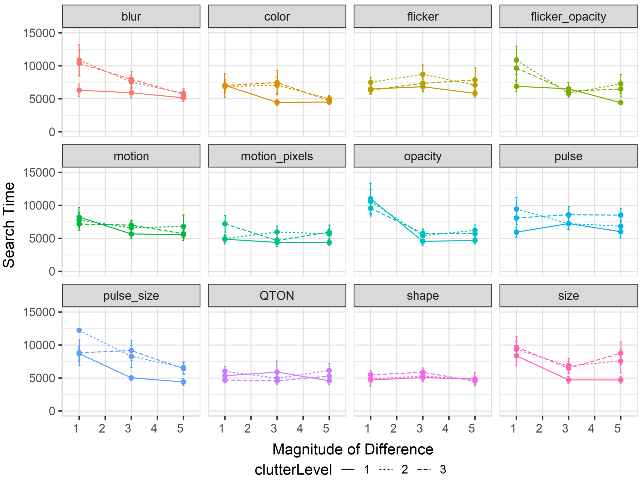

Figure 3. Experiment 2, Mean search times (±s.e.) per Variable with multiple distractor amounts. Level one (solid lines), levels 2-3 (dashed lines)

Accuracy: A 12 x 3 x 3 RM-ANOVA did not show a significant main effect of *Clutter Level* on accuracy ($F_{2,98} = 2.28, p = 0.1$), but did find main effects of *Emphasis Effect* ($F_{10,490} = 37.47, p < 0.001, \eta^2 = 0.07$) and *Magnitude of Difference* ($F_{2,98} = 262.96, p < 0.001, \eta^2 = 0.09$). The results are summarized in Fig 4.

Experiment 3: Effects of Mixed Distractor Types on Perception of Emphasis

Each participant completed 153 trials: one trial per combination of *emphasis effect* x *magnitude* x *distractor type count* (108 total), and 45 trials with no stimulus present (15 trials per distractor type count). From the 50 successful completions, the main text presents the analysis of 48 participants as two people were excluded from our analysis following a similar procedure from studies one and two. The following analysis includes all 50 completed studies.

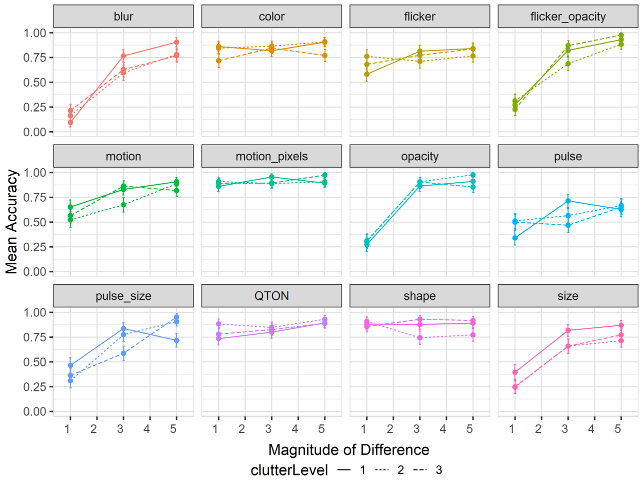

Figure 4. Experiment 2, Mean Accuracy (±s.e.) per Variable with multiple distractor amounts. Level one (solid lines), levels 2-3 (dashed lines)

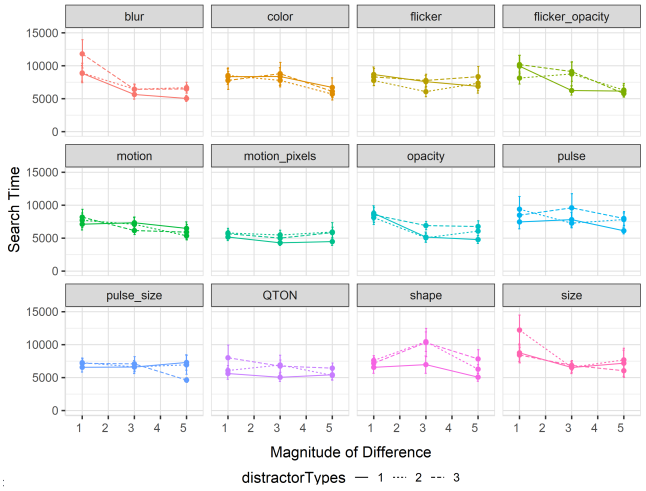

Figure 5. Experiment 3, Mean search times (±s.e.) per Variable with multiple distractor types. Level one (solid lines), levels 2-3 (dashed lines)

Results

Search Time: A 12 x 3 x 3 RM-ANOVA (*Emphasis Effect* x *Magnitude of Difference* x *Distractor Types*) showed no main effect of *Distractor Type Count* on search time ($F_{2,98} = 2.64, p = 0.07$). The results are summarized in Fig 5.

Accuracy: A 12 x 3 x 3 RM-ANOVA showed a significant main effect of *Distractor Type Count* on accuracy ($F_{2,98} = 29.41, p < 0.001, \eta^2 = 0.01$), and also showed effects of *Emphasis Effect* ($F_{11,539} = 24.40, p < 0.001, \eta^2 = 0.04$) and *Magnitude of Difference* ($F_{2,98} = 226.56, p < 0.001, \eta^2 = 0.08$). The results are summarized in Fig 6.

Experiment 4: Effects of Visualization Type on Emphasis

Each participant completed one trial per *emphasis effect* x *magnitude* x *visualization type* combination (180 total), and 15 trials with no stimulus present (5 trials per visualization

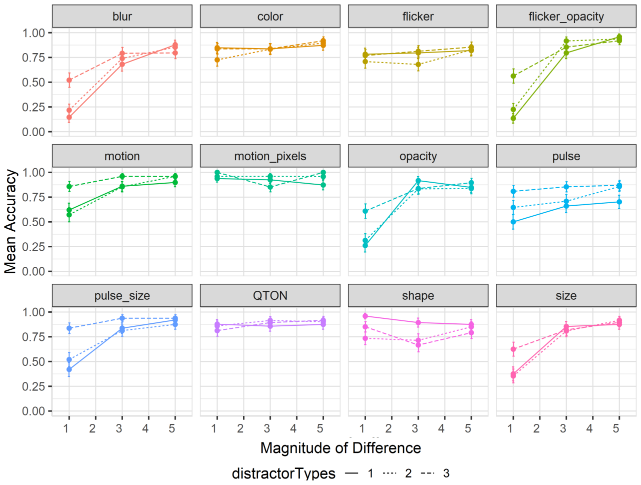

Figure 6. Experiment 3, Mean Accuracy (±s.e.) per Variable with multiple distractor types. Level one (solid lines), levels 2-3 (dashed lines)

type). From the 50 successful completions, we followed the same exclusion criteria described above, resulting in 3 participants being excluded from our analysis for for overall completion time outside 3 s.d from the mean (1) or failing engagement checks including skipping trials and rating questions (2).

Results

Search Time: A 12 x 3 x 5 RM-ANOVA (*Emphasis Effect* x *Magnitude of Difference* x *Visualization Type*) showed a significant main effect of *Visualization Type* ($F_{4,196} = 51.92, p < 0.001, \eta^2 = 0.02$) on search time. The RM-ANOVA also showed main effects of *Emphasis Effect* ($F_{11,539} = 33.57, p < 0.001, \eta^2 = 0.03$) and *Magnitude of Difference* ($F_{2,98} = 80.29, p < 0.001, \eta^2 = 0.02$). The results are summarized in Fig 7.

Accuracy: A 12 x 3 x 5 RM-ANOVA showed a significant main effect of *Visualization Type* ($F_{4,196} = 25.75, p < 0.001, \eta^2 = 0.01$) on accuracy. The RM-ANOVA also showed main effects of *Emphasis Effect* ($F_{11,539} = 81.19, p < 0.001, \eta^2 = 0.09$) and *Magnitude of Difference* ($F_{2,98} = 391.21, p < 0.001, \eta^2 = 0.08$). The results are summarized in Fig 8.

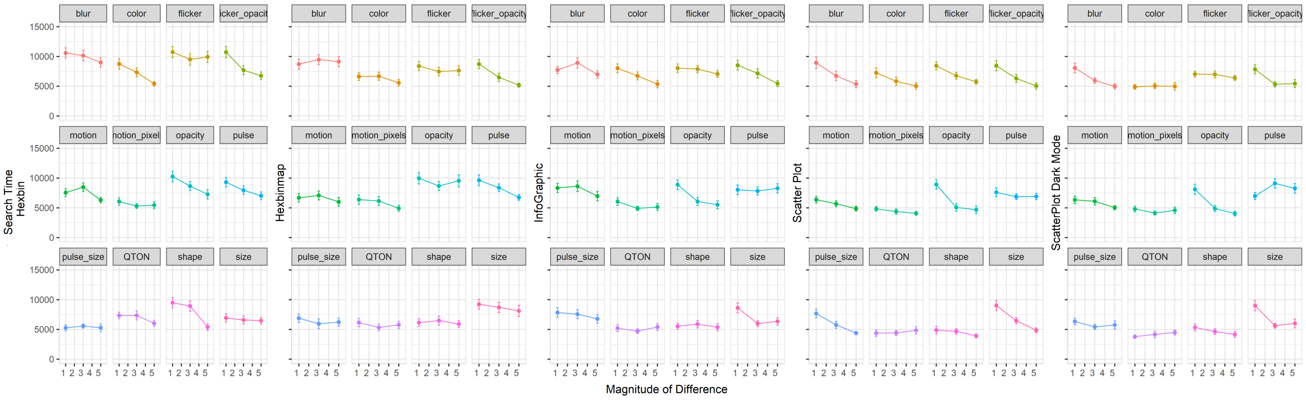

Figure 7. Mean Search Times (±s.e.) per Variable and Visualization Type.

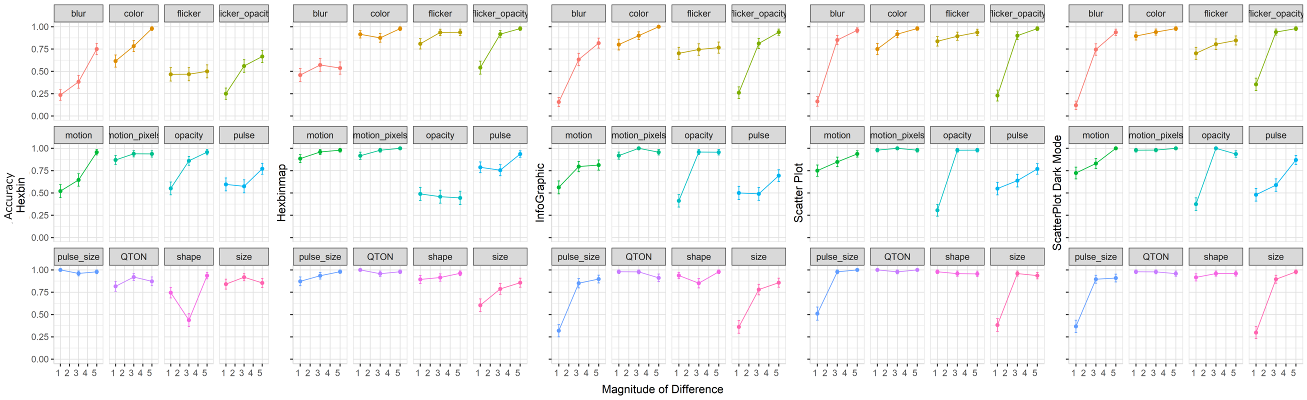

Figure 8. Mean Accuracy (±s.e.) per Variable and Visualization Type.
